# Endogenous Aβ and Exogenous Wheat Gluten Nanostructures: Understanding Peptide Self-Assembly in Disease

**DOI:** 10.1021/acsnano.5c01662

**Published:** 2025-08-08

**Authors:** María G. Herrera, Lidia Ciccone, Lara H. Moleiro, Nicolo Tonali, Verónica Isabel Dodero

**Affiliations:** † Laboratorio de Genómica e Ingeniería de Sistemas Biológicos, Instituto de Biociencias, Biotecnología y Biología Traslacional (iB3), Departamento de Fisiología y Biología Molecular y Celular, Facultad de Ciencias Exactas y Naturales, Universidad de Buenos Aires, Intendente Güiraldes 2160, Ciudad Autónoma de Buenos Aires C1428EGA, Argentina; ‡ Department Molecular Cell Biology, Institute of Biochemistry and Pathobiochemistry, 9142 Ruhr University Bochum, Bochum 44801, Germany; § 9310 University of Pisa, Department of Pharmacy, Via Bonanno 6, 56124 Pisa, Italy; ∥ Department of Physical Chemistry, 16734 Universidad Complutense de Madrid, Ciudad Universitaria s/n E28040 Madrid, Spain; ⊥ 121234 CEA Saclay, DRF/JOLIOT/DMTS/SIMoS, 91191 Gif-sur-Yvette, France; # Bielefeld University, Faculty of Chemistry, Universitätsstr. 25, 33615 Bielefeld, Germany

**Keywords:** Aβ40/42, gliadin peptides, oligomers, polyproline helix II, β-sheet, Alzheimer’s disease, gluten-related disorders, celiac disease, ataxia

## Abstract

The self-assembly of endogenous and exogenous peptides into proteolysis-resistant oligomers can trigger toxic cellular events and diseases. In Alzheimer’s disease (AD), the structural polymorphisms of endogenous amyloid-β (Aβ) 1–40 and 1–42 aggregates are essential for their neurotoxic effects. Recent findings on structural differences between brain-derived and *in vitro* fibrils underscore the need to improve the molecular and supramolecular models of diseases, for example, by stabilizing monomer conformations that lead to disease-relevant structures. In gluten-related disorders (GRDs), particularly celiac disease (CeD), research focuses on exogenous proteolytically resistant gliadin peptides (PRGPs) such as the 33-mer, p31–43, and pepsin-trypsin-derived gliadin peptides. Notably, these PRGPs form nanostructures, which may explain their behavior as nonreplicating pathogens. Thus, understanding their self-assembly has recently gained attention. This review invites both newcomers and experts in the field to tackle the challenges of characterizing peptide self-assembly process as first step to develop successful therapeutic interventions. For AD researchers, it highlights protocols for obtaining monomers and their supramolecular characterization to uncover mechanisms of brain-derived fibril formation, while also showcasing opportunities to explore PRGP nanostructures. For GRD researchers, it offers protocols to obtain PRGP nanostructures and their thorough characterization prior to cellular studies, inspired by approaches in AD research. This review contributes to interdisciplinary efforts toward therapeutic strategies grounded in molecular and supramolecular data by outlining structural insights, characterization protocols, and existing knowledge gaps. Its final aim is to connect established and emerging research domains related to Aβ and gliadin peptides that may have potential applications in peptide self-assembly and the gut-brain axis research, respectively.

## Graphical abstract

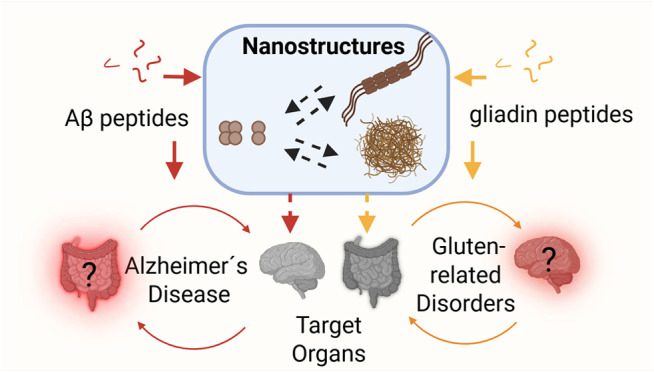

## 1 Introduction

Endogenous and exogenous peptides can undergo conformational changes and self-associate to form deposits in various organs. These peptide aggregates are resistant to enzymatic degradation in both extracellular and intracellular compartments. Endogenous peptide or protein aggregates are linked to more than 50 human diseases, including neurodegenerative disorders like prion disease, Alzheimer’s disease (AD), and Parkinson’s disease (PD), as well as metabolic syndromes like type II diabetes and other metabolic storage diseases such as Cardiac amyloidosis.^1–6^ The formation of such deposits composed of amyloid fibrils is preceded by the emergence of soluble, metastable, and transient intermediates, referred to as oligomers, and fibril-type core structures known as protofibrils.^7,8^ The amyloid fibrils observed in different pathologies share structural similarities in their quaternary structure and ultrastructure, characterized by a cross-β backbone.^9–11^ Since various aggregation species share the same process, identifying the specific nanostructure responsible for observed toxicity is often challenging. As a result, many molecular mechanisms and interactions involved in amyloid protein/peptide misfolding and aggregation remain poorly understood, limiting therapeutic intervention.^2,9^ Furthermore, the environmental triggers or cofactors that contribute to amyloid aggregation and subsequent disease development remain elusive. Representative peptides involved in amyloid-related diseases include the amyloid-β (Aβ) peptides derived from the amyloid β precursor protein (APP), which are considered the culprits in AD (Figure 1A).^12^

**Figure 1:**
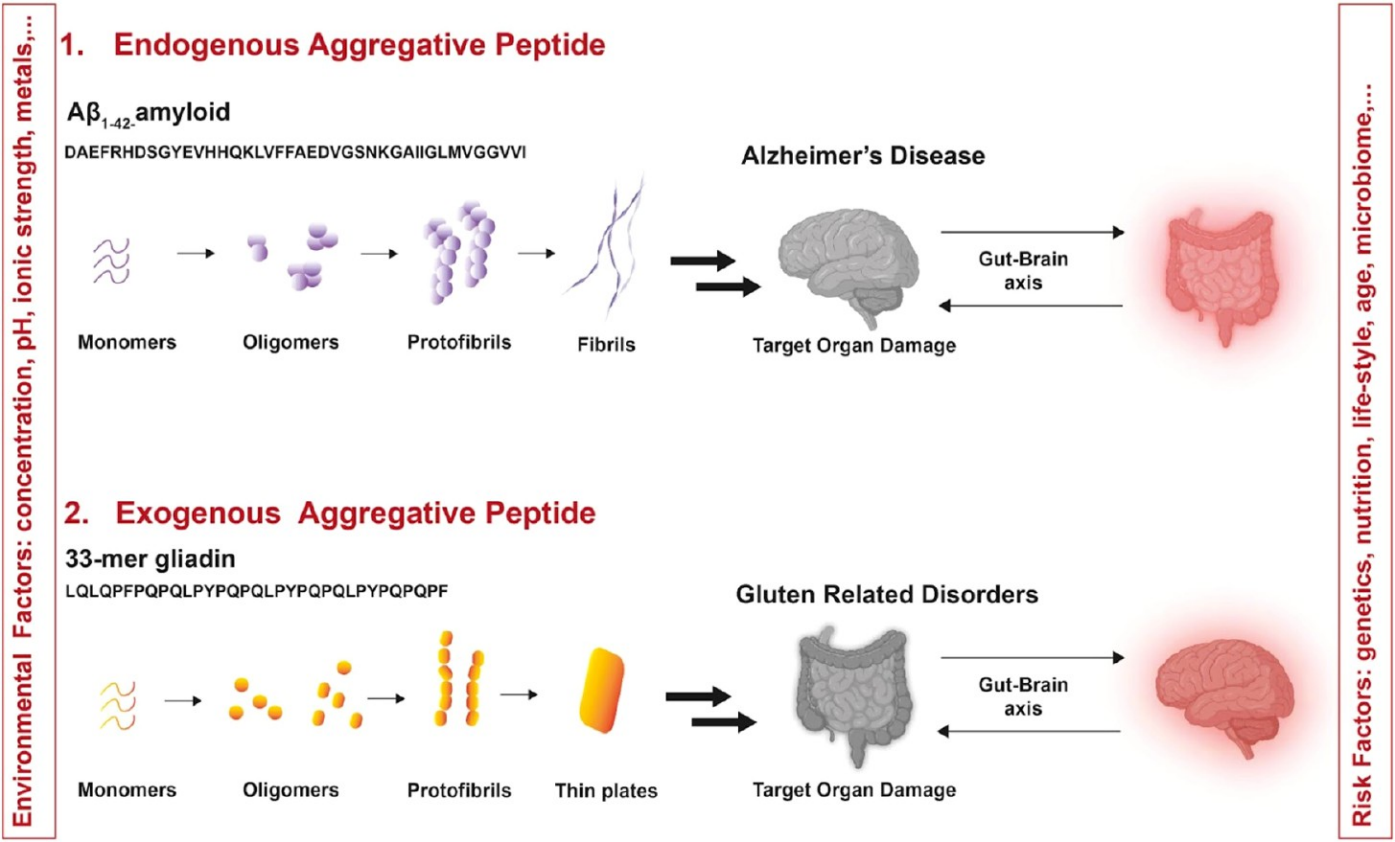
Schematic representations of the self-assembling processes of the endogenous Amyloid-β and one of the most relevant peptides in gluten-related disorders, the 33-mer gliadin peptide (33-mer). Created with BioRender.com.

Interestingly, while Aβ research has primarily focused on the brain, studies in animal models—not fully detailed here^13,14^ —suggest that peripheral Aβ oligomerization in the gut may be crucial in the early stages of AD.^13,15–19^ The gut-brain axis refers to the bidirectional communication network between the gastrointestinal tract, including its resident microbiota, and the central nervous system (CNS).^20^ This interaction is mediated through neural pathways, including the vagus nerve, as well as through the sympathetic and parasympathetic branches of the autonomic nervous system and proprioceptive fibers. This complex circuit exchanges various signaling molecules, such as neurotransmitters (e.g., serotonin and GABA), neuropeptides, cytokines, and microbial metabolites such as short-chain fatty acids (SCFAs). These signaling molecules influence the gastrointestinal physiology and immune responses, affecting brain function and disease development.^21,22^

In this context, specific exogenous proteolysis-resistant proteins and peptides derived from microbial or food proteins such as milk, egg, and gluten are investigated in connection with human diseases.^23–28^ Most proteolysis-resistant food-derived peptides can aggregate into amyloid or amyloid-like fibrils, whereas others are stabilized in prefibrillar nanostructures. Although they primarily damage the gut, many of these peptides are associated with neuroinflammation through their effect on the gut-brain axis.^29–33^ Celiac disease (CeD), the autoimmune presentation of gluten-related disorder (GRDs)^34,35^ is the most studied food-related disease triggered by exogenous, proteolysis-resistant gluten peptides. Among these, the 33-mer gliadin peptides from wheat gluten are the most studied proteolytically resistant gliadin peptides (PRGPs), known for their association with adaptive immunity in CeD. Recent findings indicate that the 33-mer gliadin peptide can form kinetically entrapped oligomers and larger structures, which may accumulate in the gut epithelium during the early stages of CeD (Figure 1B).^28,36,37^

Furthermore, many PRGPs enter the human body through the gut in individuals who consume wheat gluten, and approximately 600 PRGPs are excreted in the urine.^38^

Notably, CeD and the relatively new nonceliac gluten sensitivity (NCGS) are associated with neurological complications, with ataxia and peripheral neuropathies being the most commonly reported.^29,39–42^ Therefore, the impact of gluten on the gut-brain axis is a relevant area of research.^41,43,44^ Interestingly, deposits of immunocomplexes, primarily comprised by the enzyme transglutaminase and gluten peptides, are central to the pathomechanism of CeD; however, their ultrastructural characterization remains unexplored. Similar immunocomplexes deposits have also been identified in cases of gluten ataxia, and adherence to a gluten-free diet has been shown to improve the symptoms and prevent progression of ataxia, accompanied by a reduction in serum of antigliadin and autoantibodies.^45–47^

Additionally, recent studies have reported that dietary wheat gluten induces astro- and microgliosis in animal models.^48^ At this point, it is important to note that AD and GRDs are not directly connected;^49^ however, we believe that highlighting their similarities and differences from a chemical perspectives can contribute to develop characterization strategies and effective biological models to better understand the effects of these nanostructures beyond their primary target organs, particularly in the gut-brain axis (Figure 1).

Therefore, this review aims to highlight both Aβ peptides and PRGPs, along with their associated nanostructures, in the context of their respective pathomechanism, using molecular and supramolecular strategies. We hypothesize that contrasting current knowledge by outlining their similarities, differences, and existing research gaps will inspire new directions for future research and therapeutic approaches.

In Section 2.1, we provide a brief introduction to AD pathology, followed by the latest structural information obtained from Aβ1–40 and Aβ1–42 amyloid fibrils derived from *in vitro* experiments and patients with AD. We summarize methods for reconstitution or amplification of Aβ peptides, comparing these with those obtained through *in vitro* methods. Second, we present selected sample preparation strategies recently reported, which focus on stabilizing the monomer to control the final morphology of nanostructures, validated by multitechnique biophysical evaluations. The challenge lies in understanding and controlling the transition from monomers to oligomers and ultimately obtaining fibrils whose structural characteristics mirror those found in the brains of patients with AD. This approach will enable the design of targeted therapeutic interventions.

In Section 2.2, we shift our focus to the oligomerization of PRGPs, particularly in relation to their role in GRDs and especially CeD and NCGS with the aim to expand our knowledge of pathomechanism in GRDs. As structural evaluation in this field is still in its early stages, we present all the investigations about oligomers and fibrils formed by PRGPs that correlate with biological information. We describe the 33-mer gliadin nanostructures, as well as other less-studied PRGP oligomers and fibrils, derived from the synthetic p31–43 peptide and those formed during gliadin digestion by pepsin and pepsin-trypsin. Much of our current understanding of PRGP self-assembly is based on methodologies inspired by amyloid peptides, such as Aβ peptides.

Section 3 aims to provide similarities and differences, as well as research gaps. Among them, the most relevant differences are that under physiologically relevant conditions, synthetic pure gliadin peptides form kinetically trapped oligomers and protofibrils that do not progress into fibril amyloids—at least in the tested conditions—whereas heteroassemblies of gliadin peptides spontaneously form amyloid-like fibrils^37,50,51^ In contrast, Aβ oligomers can evolve into cross-β rich amyloid fibrils within minutes to days, depending on experimental conditions. A notable feature in GRDs is that adherence to a lifelong gluten-free diet can reverse the pathology, highlighting the direct relationship between the PRGPs intake and GRDs.^52^ This reversibility contrasts with AD, considering that Aβ is an endogenous peptide. Finally, Section 4 aims to summarize new research areas for understanding and addressing the molecular mechanisms underlying the self-assembly of these peptides that could bridge current knowledge gaps and foster innovative research directions in peptide self-assembly with clinical biomedical implications.

## 2 From Alzheimer’s Disease to Gluten-Related Disorders: A Supramolecular Perspective on Peptide Self-Assembly

### 2.1 AD and Aβ Peptide Aggregates

AD can be clinically divided into sporadic (SAD) and familial (FD) AD.^53^ FD accounts for 1–5% of all AD cases and most are associated with mutations in three genes (APP, PSEN1, and PSEN2). Additionally, more than 20 genetic risk loci for AD have been identified, including the ε4 allele of the apolipoprotein E (APOE). The APOE4 genotype influences both the timing and amount of amyloid deposition in the human brain.^54^ Conversely, the incidence of SAD accounts for more than 95% of all AD cases. Different hypotheses have been proposed to explain the cause of SAD, including the cholinergic, amyloid, tau propagation, inflammatory, neurovascular, metal ion, and lymphatic system hypotheses.^55^ For over 30 years, the amyloid cascade hypothesis has been the dominant framework for understanding AD pathogenesis. First proposed in the early 1990s, it suggests that the accumulation and aggregation of Aβ peptides trigger a cascade of neurotoxic events, including tau pathology, neuroinflammation, synaptic dysfunction, and, ultimately, neurodegeneration. Decades of research have provided strong genetic and biochemical evidence supporting this, particularly through studies on familial AD mutations in APP, PSEN1, and PSEN2, which directly influence Aβ production and aggregation.^8^ Despite challenges, the amyloid hypothesis continues to shape therapeutic strategies, with recent advances in anti-Aβ immunotherapies offering renewed hope. Recently, the β Amyloid Dysfunction (BAD) hypothesis focused on the role of Aβ oligomers, considering them as early transient species that form through dysregulations of Aβ homeostasis and interfere with the synaptic physiological Aβ monomer regulation.^56^ Hence, oligomers represent soluble misfolded aggregates that appear early during the aggregation process and undergo a structural transformation into dimers or multimers. In contrast to fibrils, misfolded oligomers isolated from the brain *in vivo* dissolve in SDS-containing buffers, enabling them to be distinguished from smaller fragments with a fibril-type core structure known as protofibrils, which are often mistaken for oligomers.^56^ Thus, monomeric Aβ can be considered a benign preamyloid peptide serving as a permanent source for an essential physiological cofactor in the synaptic vesicle cycle process.^57^

Hence, Aβ oligomers show synaptic toxicity via potential receptor-mediated effects,^58^ in addition, they can be considered an early sign of Aβ monomer misfolding that leads to a deficiency of Aβ monomer at the synapse.^56^

Although the aggregation pathways and species have been characterized *in vitro*, their effects in the development of Aβ proteinopathies are poorly understood. Briefly, soluble Aβ oligomers have been implicated in cellular membrane disruption, dysregulation of ion transport,^59^ pathological activation of membrane receptors,^60^ and oxidative stress,^61^ accompanied by the phosphorylation of AD-related target proteins.^62^ In addition, post-translational modifications of Aβ peptides have been linked to enhanced cytotoxic effects.^63^

Several studies have highlighted the role of the microglia and astrocytes reacting toward Aβ species, which induces the secretion of inflammatory molecules.^64,65^ Aβ oligomers have also been associated with the activation of advanced glycation end products (RAGEs), Toll-like receptors (TLRs), and NOD and TREM receptors.^66^ Soluble Aβ oligomers promote Toll-like receptor 4 (TLR4) sensitization in microglia and astrocytes, increasing TNF-α production. Furthermore, these oligomers impair long-term potentiation (LTP) in hippocampal slices in a mice model and trigger neuronal cell death in astrocyte-neuron cocultures, effects that are prevented by TLR4 antagonists.^67^ Notably, Aβ fibrils are recognized by TLR2, promoting an inflammatory cascade in mice models.^68^ Moreover, the presence of Aβ oligomers induces an increase of monocytes, macrophages, and dendritic cells in the brain of patients with AD, along with a decrease in resting NK cells.^69^ The accumulation of these immune cells, which are required to clear Aβ oligomers and aggregates, induces oxidative stress, exacerbates disease pathology, and promotes peptide modifications that further enhance aggregation.^70,71^

In patients with AD, microglial cells react to Aβ by increasing inflammatory biomarker levels, such as cytokines, which contribute to synaptic destruction and neuronal death.^72,73^

Additionally, other humoral factors such as complement components (1q, predominantly and in minor proportion C3 and C4) are increased in AD, suggesting that hyperactivation may contribute to disease progression.^74^ Nevertheless, whether there is an accumulation of inflammatory biomarkers and then oligomerization or if the oligomerization occurs first is a matter of debate.^75^

Moreover, changes in the brain microenvironment lead to increased permeability of the blood-brain barrier,^76^ facilitating lymphocyte T infiltration, suggesting their participation in the response in AD.^77^ Among T cells, the cytotoxic CD8+ are the most abundant, whereas regulatory T cells are reduced.^78^ Interestingly, Aβ-specific T cells have been shown to produce high levels of IFN-γ, which increases CD11b expression—an integrin subunit involved in cellular adhesion and Aβ deposition.^79^ However, the study of Gericke et al. shows that Aβ1–42 oligomers inhibit antigen presentation and the response of CD4+ T cells *in vitro*. Specifically, Aβ1–42 oligomers reduce T cell activation and division measured by the expression of IFNγ and the proliferation marker *K*_i_-67.^80^ Nevertheless, the direct contribution of T cells to n-ins unclear. Regarding the adaptive humoral response, antibodies targeting the N-terminal region of Aβ have been detected in some cases of severe AD.^69^ Dalgediene et al. analyzed the humoral immune response in mice immunized with different Aβ1–42 oligomers, finding that smaller oligomers (1–2 nm) induced a stronger immune response than that of larger oligomers or monomers.^81^ The role of B cells in AD pathology is also controversial, as some subsets promote the secretion of pro-inflammatory mediators, whereas regulatory B cells may help mitigate immune activation.^82^

Although multiple mechanisms have been described in the immune response to Aβ peptides, the presence of Aβ-specific CD4+ and CD8+ T cells has not been detected in brain samples from patients with AD. These studies suggest a complex and nonlinear activation of the immune response in AD and the function of Aβ-specific T cells in neuroinflammation, Aβ accumulation, and cytotoxic responses, which remain poorly understood.^83^

For more detailed information on the pathomechanism, please refer to the following reviews of Iliyasu et al., Isu et al.,^84–86^ and Figure 2.

**Figure 2:**
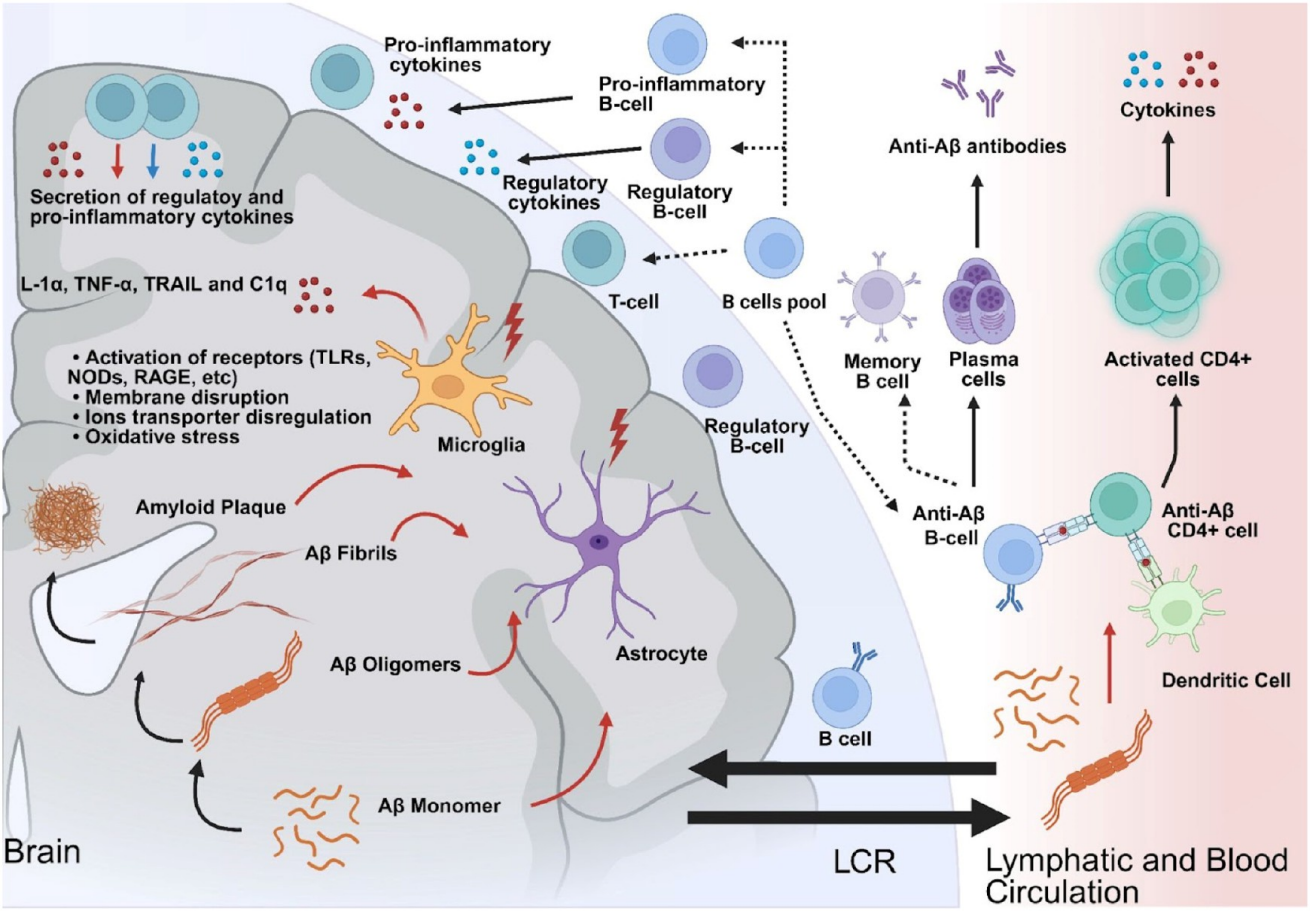
Findings and hypotheses on the role of the immune system in AD pathology. Different structural forms of Aβ may activate and stress the microglia and astrocytes through different mechanisms, leading to the production of several cytokines and pro-inflammatory molecules. Soluble monomers and oligomers can enter the lymphatic and blood circulation and may stimulate an innate immune response. This is suggested by molecular signatures in monocytes, macrophages, and dendritic cells, along with reduced levels of resting NK cells in brain samples from patients with AD. The classical adaptive immune response has also been observed, including the production of Aβ antibodies and inflammatory cytokines with a CD4+ profile, leading to the generation of memory B cells. Interestingly, neuroinflammation can also be increased by the activity of B cells, whose cell pool seems to be heterogeneous. These cells may present different cytokine profiles: one that contributes to producing pro-inflammatory cytokines and another inducing anti-inflammatory response. Additionally, autoantigen-specific B cells can stimulate T cells by presenting Aβ, an autoantigen, in complex with MHC molecules, promoting a selective response toward Aβ. The B cell-dependent activation of T cells can occur in lymphoid organs or in the brain parenchyma. B cells may further contribute to AD progression by hypersecreting pro-inflammatory mediators, which enhance microglia activation and neuroinflammation in the brain. On the contrary, active T cells and some CD8+ effector memory T cells accumulate in the brain and cerebrospinal fluid of patients AD, suggesting their contribution to AD. Moreover, CD4+ T cells interact with microglia, promoting or impeding the development of AD through cytokine signaling. Despite these findings, Aβ-specific CD4+ and CD8+ T cells have not yet been reported in AD brain samples. Notes: red, blue, and dashed arrows indicate stimulatory, regulatory, and hypothetical pathways, respectively.^83^ Adapted with permission under Creative Commons CC BY 4.0 license from ref 83. Copyright 2024 The Authors. Published by MDPI. Created with BioRender.com.

#### 2.1.1 Role of Aβ Nanostructures in AD

Aβ protein fibrillar species have long been considered AD’s most toxic aggregate forms. Aβ peptides derive from the APP transmembrane glycoprotein, which is cleaved by β-secretase and γ-secretase to release the amyloid β-peptide containing 40 and 42 amino acids, denoted as Aβ1–40 or Aβ1–42, respectively.^12^ Both peptides represent the main component of senile plaques, which are classical neuropathological hallmarks of AD. The fibrillar architecture of the amyloid plaques has been elucidated, especially in the past few years with the advent of cryo-electron microscopy (cryo-EM).^87–90^ However, these aberrant and late-stage aggregates result from a more complex mechanism of aggregation, which is characterized by early intermediate and metastable soluble oligomers. These early nanostructures are often considered more toxic than mature fibrils, although their properties such as hydrophobicity, size, and degree of metastability, are less characterized due to their elusive and heterogeneous nature.^91^ Aβ peptides are not inherently resistant to proteolysis, as they are degraded by various proteases, including neprilysin, insulin-degrading enzyme (IDE), and other extracellular and intracellular proteases.^92^ However, once Aβ aggregates into oligomers, fibrils, or plaques, they become highly resistant to proteolysis because of their β-sheet-rich structure, which sterically hinders proteases access to cleavage sites. Aβ is an intrinsically disordered peptide (IDP) ranging from fully unstructured to partially structured, including random coil, molten globule-like aggregates, or flexible multidomain conformations (Figure 3). Moreover, it contains multiple local energy minima separated by low energy barriers, spacing from the α helix, polyproline II (PPII)-helix, and β-hairpin in different combinations and compositions.^93^ PPII-helix is an extended conformation, possibly facilitated by steric interactions between bulky side chains. The dihedral angles in the PPII-helix are very close to those of an aggregation-prone β-conformation. The transition to this conformation may lead to misfolding, while an α helix-containing conformation keeps the monomer in a nonamyloidogenic state^94,95^ (Figure 3).

**Figure 3:**
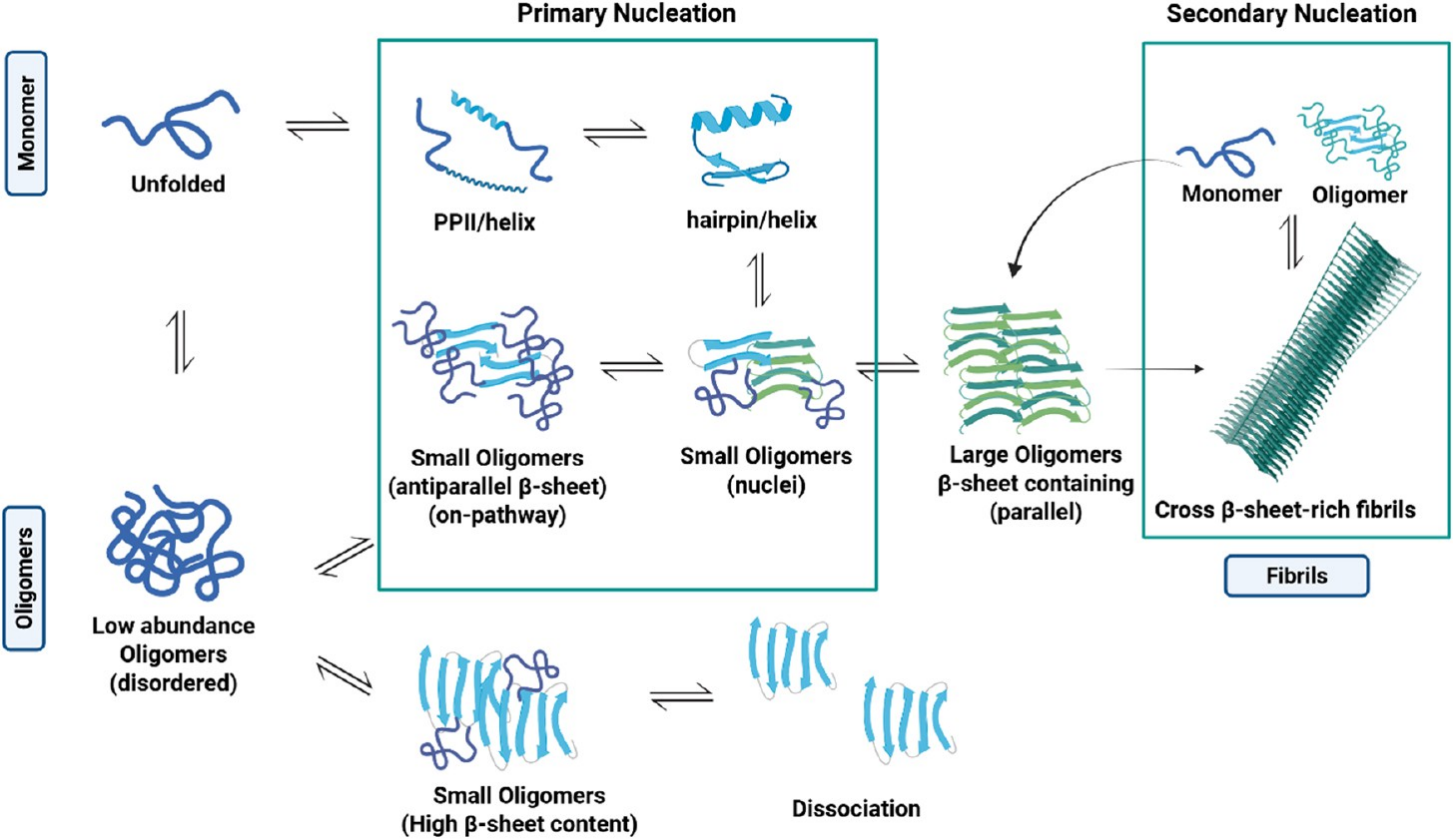
Schematic representations of the amyloid-β peptide aggregation. From the unfolded monomer state to the β-sheet-rich fibrils, passing through transient and heterogeneous oligomers. The complexity of the system is highlighted by distinguishing on- and off-pathway oligomers that either lead to or deviate from amyloid formation. The two nucleation mechanisms of oligomer formation—primary and secondary—are also illustrated. The different conformational states are shown, ranging from the monomer (PPII/helix and Hairpin/helix) to the antiparallel and parallel β-sheet conformations within oligomers and protofibrils. Created with BioRender.com.

As previously mentioned, several hypotheses have been proposed to explain the etiopathogenesis of AD. However, most of these have been recently questioned, particularly the amyloid cascade hypothesis, due to the failure of phase 3 clinical trials employing therapeutic agents targeting the amyloid pathway, which showed no cognitive improvement.^96^ The amyloid hypothesis is increasingly being questioned by many scientists, investors, and patients, though several critical issues could explain the failure of these clinical trials. One key issue is the need for high experimental reproducibility and stringent control, particularly regarding well-defined monomeric solutions with minimal seeding or chemical modification. This lack of reproducibility has often led to researchers reporting inconsistent kinetic profiles.^97^ The absence of a consensus on how to achieve a rational reconstitution protocol for Aβ peptides that guarantees reproducible aggregation kinetics casts doubt on the validity of many results regarding the assessment of agents that inhibit aggregation and neurotoxicity, as well as the characterization of the diverse minor and transient assemblies typically considered neurotoxic.

#### 2.1.2 Aβ Fibrils from Patients with AD

Aβ fibrils, composed of Aβ1–40 and Aβ1–42 peptides, are the most abundant protein-related element in the brain of patients with AD. Experimental evidence suggests that structural differences in Aβ fibrils could reflect various clinical and pathological AD phenotypes;^98,99^ thus, the structural study of Aβ fibril polymorphs has gained attention. For many years, the information on Aβ peptides has come from the studies conducted on Aβ fibrils and other forms of aggregates prepared *in vitro* using synthetic or recombinant expressed Aβ peptides. This approach has been considered a valuable alternative to human samples because Aβ aggregates generated in tubes and those fibrils purified from patient’s tissues are rich in β structures and react similarly with Congo red.^100–103^ However, Aβ fibrils prepared in tubes are characterized by a high polymorphism related to the various peptide variants used for their preparation or different fibrillation protocols. Recently, several cryo-EM structures of Aβ filaments (1–40 and 1–42) derived from the brain of patients with AD have been reported.^90,104^ Interestingly, the structures of AD brain fibrils differ significantly from those prepared *in vitro*. In the paragraphs below, the structural characteristics of the *in vivo* Aβ filaments obtained from patients with AD will be summarized and compared with the most similar *in vitro* ones.

The principal Aβ inclusions in the parenchyma and vascular deposits can be classified as diffuse or focal deposits. Diffuse deposits are characterized by poorly organized Aβ filaments in various brain regions. In contrast, focal deposits possess a spherical core of well-packed Aβ peptides surrounded by less densely packed filaments, typically located in the hippocampus and the central cortex. As AD progresses, both diffuse and focal deposits can be found throughout the brain, especially in the walls of blood vessels.

In 2019, Kollmer et al. purified Aβ fibrils from the meninges of three patients with AD and investigated them using cryo-EM;^87^ they observed polymorphic fibrils consisting of similarly structured protofilaments (Table 1).

**Table 1:** Structural Characteristics of Aβ Protofilaments Obtained from the Brain of Patients with AD and In Vitro Amplification^a^ ^,^ ^b^

peptide	fibrils obtained by	N° patients	morphology	data availability	refs
Aβ1–40	Extraction meningeal tissue Aβ1–40 > 1–42	3	**I** width 7.4 ± 0.4 nm, crossover distance 41.5 ± 2.3 nm (Figure 3A)	EMD-10204	^87^
			**II** width 12.3 ± 0.6 nm, crossover distance 129.0 ± 10.5 nm (similar to **I** with 2 protofilaments)	PDB 6SHS	
			**III** width 17.9 ± 0.6 nm, crossover distance 142.8 ± 12.3 nm (similar to **I** with 3 protofilaments)	EMD-4864	
			All with C-shaped are right-hand twisted.	EMD-4866	
Aβ1–40	Extraction of cortical tissue + Seeds with recombinant peptide or + synthetic peptide (10 mM PBS with 0.01% NaN_3_ and pH 7.4, 24 h)	2	**I** width 107 ± 20 nm, crossover distance 120 nm	EMD-21501	^105^
			S-shaped are left-hand twisted, (Figure 3B).	PDB 6W0O	
Aβ1–40	Extraction of meningeal tissue + seeding *in vitro* with recombinant peptide seeded by ex vivo fibril (100 mM PBS pH 7.4, 24 h)	2	**I** seeded width 7.4 ± 0.4 nm, crossover distance 380.0 ± 3 nm	EMD-17166	^106^
				PDB 8OT1	
			C-shaped right-hand twisted	EMD-17167	
				PDB 8OT3	
			**I** and **II** unseeded width indistinguishable, crossover distance 108 ± 8 and 80.0 ± 4 nm, respectively left-hand twisted.	EMD-17168	
				PDB 8OT4	
Aβ1–42	Sarkosyl extraction from the brain	10	**I** and **II** have an S-shaped right-hand twisted	EMD-13800	^90^
				PDB 7Q4B	
			Protofilament **I** is packed with pseudo-2_1_ symmetry, while Type **II** is packed with C2 symmetry, (Figure 3C,D, respectively).	EMD-13809	
				PDB 7Q4M	
Aβ1–40	Water or sarkosyl extraction from leptomeningeal tissue	3	**I** and **II** have C-shaped right-hand twisted	EMD-18508	^104^
				PDB 8QN6	
			Protofilament **I** is packed with pseudo-2_1_ symmetry, while Type **II** is packed with C2 symmetry, (similar to **I** and **II** described by Kollmer et al. Figure 3A).	EMD-18509	
				PDB 8QN7	

a Classifications I, II, and III are arbitrary divisions made by the authors of the respective articles to describe the different types of fibrils in their specimens. Therefore, it was implemented here, and it is worth mentioning that it is arbitrary.

b Width and crossover distance are parameters useful for describing amyloid fibril morphologies. Width corresponds to the lateral fibril extension, and crossover distances are apparent constrictions of the fibril width when visualizing the fibrils with transmission electron microscopy (TEM).

Then, their results were compared with all structural data available for the fibrils of Aβ produced *in vitro*;^87,88^ the *in vitro* and *in vivo* brain-derived fibrils showed a twisted morphology. However, a right-hand twist was observed only in the fibrils of patients with AD (Table 1), while a left-hand twist in the Aβ fibrils was detected *in vitro*. Three polymorphs were detected on Aβ1–40 brain-derived fibrils (Table 1): morphology I was characterized by two peptide stacks arranged with a pseudo 2_1_-screw axis, with four cross-β sheets (β1-β4) for each protofilament; the peptide is C-shaped where the N- and C- terminal end as arches (Figure 4A), and a conformation different from that previously reported for Aβ prepared *in vitro* (we address the reader to the accurate comparison made by Kollmer et al.^87^). Morphology II and III are very similar to that of polymorph I; the main difference is in the number of protofilaments, with two or three, respectively.

**Figure 4:**
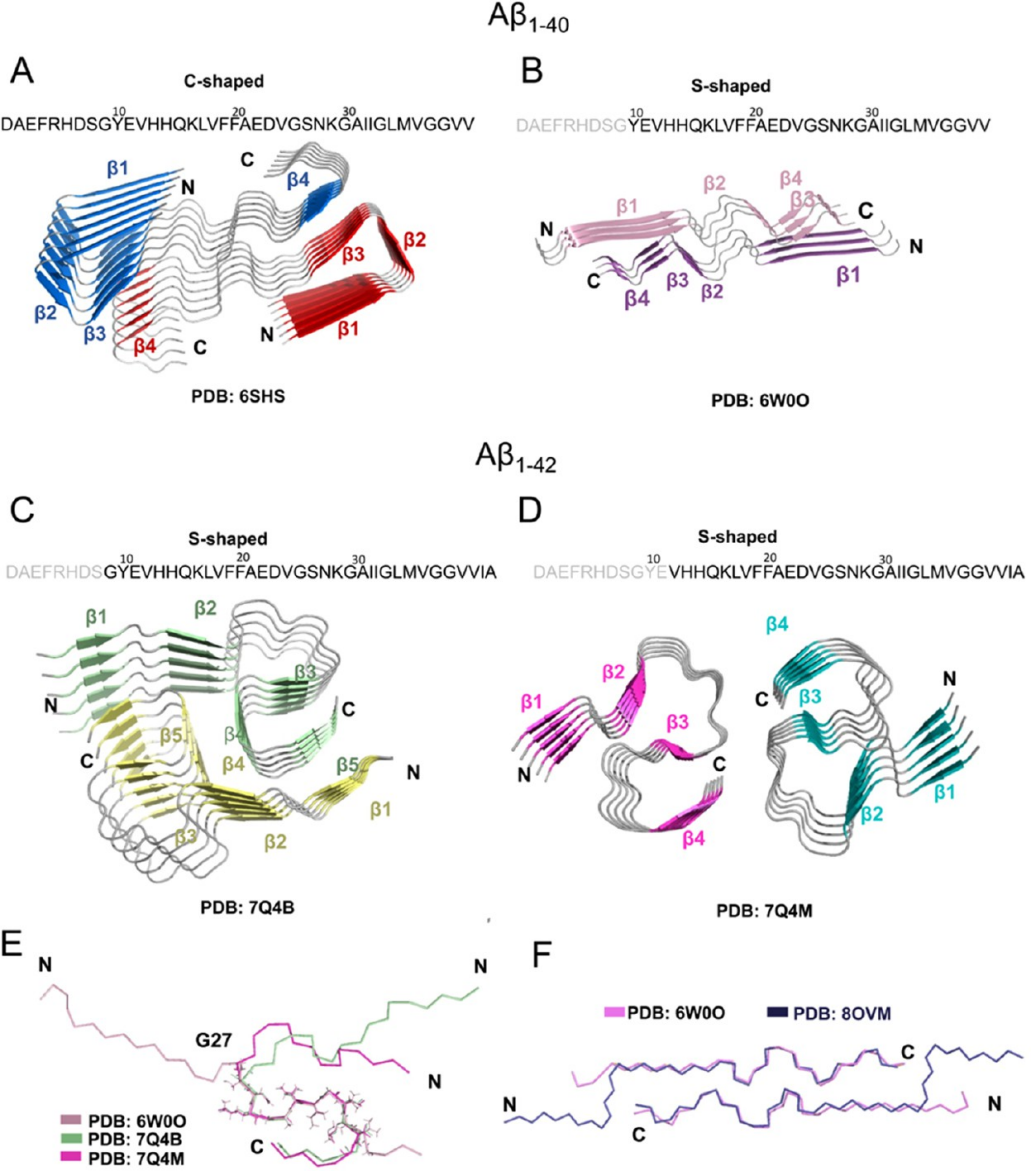
Structures of Aβ1–40 or Aβ1–42 protofilaments. (A) Aβ1–40 fibrils extracted from meningeal tissue of patients with AD. (B) Aβ1–40 fibrils amplified in vitro by seeds from fibrils extracted from the cortical tissue of patients with AD. (C, D) Aβ1–42 fibrils extracted from the brains of patients with AD: (C) Type I and (D) Type II. (E) Superposition of S-shaped fibrils extracted from the human brain (PDB 7Q4B and 7Q4M) with brain-derived S-shaped fibrils amplified in vitro (PDB 6W0O). (F) Comparison between brain-derived amyloid fibrils amplified in vitro and those grown in the presence of lipids. Created using the PyMOL program.^115^

Experimental evidence suggests that Aβ fibril polymorphisms may be associated with different pathological features in patients with AD. This could explain the structural discrepancies observed between the various Aβ fibril polymorphs extracted from human brains and those prepared using *in vitro* protocol.^87,88,107^ In this context, the study of Aβ1–40 and Aβ1–42 fibrils obtained through seeded growth from AD brain extracts has been proposed as an alternative approach to shed light on Aβ fibril polymorphism. A particularly interesting result came from Qiang et al., who identified the NMR spectroscopic signatures of the most prevalent Aβ1–40 fibril polymorph in AD brains by analyzing fibrils amplified by seeds derived from the cerebral cortical tissue of patients with AD.^108^ In 2021, the structure of this polymorph was studied in detail using cryo-EM.^105^ The high-resolution density map (2.8 Å) revealed that the fibrils exhibited a four-layered cross-β structure with 2-fold rotational symmetry in the repeat unit and a 2_1_-screw symmetry with a left-handed twist^105^ (Table 1). The density map was well aligned with the amino acids between residues 13 and 40, where residues 13 to 22 form a long N-terminal β-strand and additional β-strands are located in the C-terminal region between residues 30–32, 34–36, and 38–39 (Figure 4B). Unlike previously reported structures, each Aβ1–40 filament in this polymorph is almost entirely extended. This central core is surrounded by an unresolved density β-cross motif, which, as suggested by the authors, may be linked to a secondary nucleation process.^105^

The effect of seeding recombinant Aβ peptides with brain-derived Aβ fibrils *in vitro* was also investigated by Pfeiffer et al.^106^ They hypothesized that seeds from patients with AD could influence the growth of the *in vitro* Aβ fibrils, promoting specific structural characteristics.^106^ The study found that fibrils extracted from the meninges of two patients with AD could seed recombinant Aβ peptides, resulting in the first generation of fibrils primarily exhibiting morphology I, as previously reported by Kollmer et al.^87^ (Table 1). Interestingly, the data suggested that the structures of fibrils extracted from patients with AD promote different morphologies across successive generations of fibril amplification. While the first generation of fibril growth, seeded with brain-derived fibrils, produced right-handed twisted fibrils, the third generation exhibited a left-handed twist, confirming previous observations by Ghosh et al. (Table 1). This study reinforced the high variability and structural differences between fibrils grown *in vitro* and those derived from the brain. It also suggested that even when fibrils are extracted from patients with AD, the protocols used for fibril seed amplification and dilution across generations can influence the morphology, leading to new nucleation events and different fibril structures.^106^

Another critical study was performed by Yang et al.; they solved the structures of Aβ1–42 fibrils extracted from the brains of ten patients, five with AD and five affected by aging-related diseases, using the sarkosyl method,^90,109^ wherein types I and II morphologies were identified (Table 1). Type I was characteristic of the brains of patients with sporadic AD, while type II was found in cases with diffuse deposits and focal Aβ plaques. From a structural point of view, type I Aβ1–42 filaments are formed by two S-shaped peptides folded around a pseudo 2_1_ symmetry and a secondary structure characterized by five β strands (Figure 4C).^90^ Type II shows an S-shaped fold, but the orientations of some amino acid side chains differ, inducing a twist characteristic of type II. The two protofilaments had a C2 symmetry (Figure 4D).^90^ Type I and II filaments showed a left-handed twist and appeared structurally different from the Aβ1–40 fibrils extracted from the meninges of patients, which have a C-shaped fold and a right-handed twist^87^ (Table 1 **)**.

In the central core, between G25 and G37, the amino acids of type I and II filaments share a similar disposition previously reported for the amino acids of the Aβ1–40 structure determined using *in vitro* fibrils seeded from the cerebral cortex of patients with AD^105^ (Figure 4E). However, despite this similarity, type I and II protofilaments are different from previous left-handed Aβ1–40 fibrils.^105^ From their structural analysis, Yang et al. reported that although the fibrils initially appeared similar to those formed *in vitro* (all had S-shaped domain), a more detailed investigation revealed differences in the orientation of amino acids side chains.^88,90,110–112^ In contrast, similarities were detected between the *in vitro* filaments Aβ1–40 with the Osaka mutation and type II Aβ1–42 fibrils.^90,113^

Recently, high-resolution (up to 2.4 Å) Cryo-EM structures of Aβ1–40 filaments extracted from the leptomeninges of three brains of patients with AD have been reported.^104^ Water and sarkosyl extraction protocols were conducted, obtaining the same results. In agreement with previous reports, three types of C-shaped right-handed Aβ1–40 filaments were found, with one, two, or three pairs of protofilaments.^87,104^ Type I was characterized by a pair of protofilaments packed with pseudo-2_1_ helical symmetry, while Type II exhibited C2 symmetry with two pairs of protofilaments running side by side. Both types exhibited a right-handed twist (Table 1). Interestingly, the structural analysis supports the hypothesis that Aβ1–42 deposits in parenchymal deposits and blood vessel deposits are distinct, suggesting that AD and cerebral amyloid angiopathy represent different Aβ proteinopathies,^104,114^ however, the sampling was small, and the information about patients is limited.

Given the structural data reported in this section, it is evident that, while polymorphism is an intrinsic feature of Aβ deposits, the Aβ protofilaments prepared *in vitro* do not perfectly match the morphologies of Aβ fibrils extracted from the brains of patients with AD. However, it would be premature to conclude that *in vitro*-formed fibrils are unequivocally different from those extracted from patients with AD. Indeed, considering the hypothesis that AD is linked to specific fibril morphologies, the design of inhibitors targeting these fibrils is essential. Therefore, improving *in vitro* fibril formation protocols to better mimic the *in vivo* environment could provide an effective strategy for the specific inhibition of amyloid fibrillization.

In this context, a fascinating work has been recently published by Frieg et al.,^89^ wherein they grew Aβ1–40 fibrils in the presence of lipid vesicles, finding that the *in vitro* fibrillization with lipids induced a fold similar to that of brain-seeded fibrils.^89^ Recombinant monomeric Aβ1–40 was diluted in 10 mM of PBS at pH 6.5 in the presence of lipidic vesicles for 1–2 days at 37 °C and the structures of the fibrils were solved by cryo-EM. Three filaments folded and six polymorphs were found, all containing lipids. The most abundant was polymorph I, characterized by two intertwined protofilaments of the same fold with a pseudo 2_1_ screw symmetry (Table 1). The comparison between this polymorph and the previously reported structure of a brain-derived fibril of patients with AD amplified *in vitro* showed that they were almost identical^105^ (Figure 4F).

#### 2.1.3 Protocols to Control the Monomerization of Aβ and to Provide a Relevant End-Point Fibrils Morphology

When studying the formation of aggregates in a test tube, it is well-established that the conditions used to prepare the oligomers impact their shape and morphology. Several structural models have been proposed for Aβ oligomers, typically represented as parallel β-strands, while a high degree of structural variability exists in the supramolecular folds, which appear to be strongly influenced by the protocols used to induce specific conformations.^116^ It is important to note that the entire aggregation process and the rates of underlying steps are undoubtedly affected by solution conditions, including ionic strength, pH, and temperature.^117–119^ These factors can change the heterogeneity of the studied system in terms of pathways and structures, leading to significant changes in the kinetics, content, and morphology of the aggregates formed. Furthermore, a crucial step is isolating the aggregates from the in vitro solution or isolating the oligomers from brain tissue. In both cases, the samples may not be representative due to the formation of artifacts caused by the purification procedure.^120,121^ Over the years, significant efforts have been made to obtain information on the structural characteristics of Aβ oligomers. Despite the progress, only one atomic structure has been solved for in vitro Aβ oligomers in membrane-mimicking environments.^122–124^

Recent studies have demonstrated the pivotal role of chemical model systems in unraveling the heterogeneous structures of amyloid oligomers, providing unprecedented insights at the atomic level. These model peptides are designed to replicate the biological and biophysical characteristics of native amyloid oligomers, offering distinct advantages. Unlike native oligomers, oligomers formed by these systems typically exhibit greater homogeneity and stability, enabling more precise and detailed characterization. Kreutzer et al. described the X-ray crystallographic structure of oligomers formed by a peptide derived from residues 24–29 corresponding to a loop of Aβ1–42. They discovered that three β-hairpin monomers assemble to form a triangular trimer, and four trimers assemble in a tetrahedral arrangement to form a dodecamer. Furthermore, five dodecamers pack together to form an annular pore. This study demonstrated that identifying the minimal modification required to crystallize Aβ peptides will ultimately allow the crystallization of larger fragments of Aβ, providing significant insights into the structures of Aβ oligomers.^125^ Kreutzer et al. highlighted that in water, the macrocyclic β-sheet peptide forms hydrogen-bonded dimers that assemble to form tetramers through hydrophobic interactions. The different morphology observed between the solution- and solid-state tetramers could offer insight into the structural foundations of polymorphism among Aβ oligomers in AD.^126^ Two recent reviews emphasized the utility of these model systems in facilitating the development of chemical probes and inhibitors that target specific oligomeric species, bridging the gaps in structural knowledge of amyloid oligomers.^125,127^

The great challenge in achieving high-resolution structural information for oligomers is their dynamic and transient nature, which makes them extremely difficult to work with. Despite several available protocols, these samples are typically characterized by high heterogeneity. A thorough review of the Aβ structural models reported in the literature was published by Chen et al., with a specific section dedicated to oligomers.^116^ We kindly direct readers to this work for an exhaustive overview. One recent study by Ciudad et al. described the architecture of the Aβ oligomer formed in a membrane-mimicking environment under conditions close to biological ones. The researchers introduced a protocol to prepare β-sheet pore-forming Aβ1–42 called βPFOsAβ1–42, where Aβ1–42 adopts a specific structure and is incorporated into membranes as pores.^123^ Using NMR analysis, two different Aβ1–42 subunits were detected in the asymmetric dimer unit: one subunit consisted of two β-strands (β1 and β2), and the other consisted of a β-strand (β3) and a small portion with an α-helical aspect (α1), (Figure 5A). Unlike previous parallel models, the βPFOsAβ1–42 tetramer adopts an intermolecular antiparallel β-sheet architecture. Despite this, there is a structural similarity in the connecting loop of the β-hairpin topology between residues Asp23 and Lys28 (Figure 5A). Thus, the tetramer is composed of a β-sheet core formed by six β-strands, which are linked by two β-turns (β1-β2 and β1′-β2′) and two flexible N-termini in the middle of the asymmetric unit (β3-β3′).

**Figure 5:**
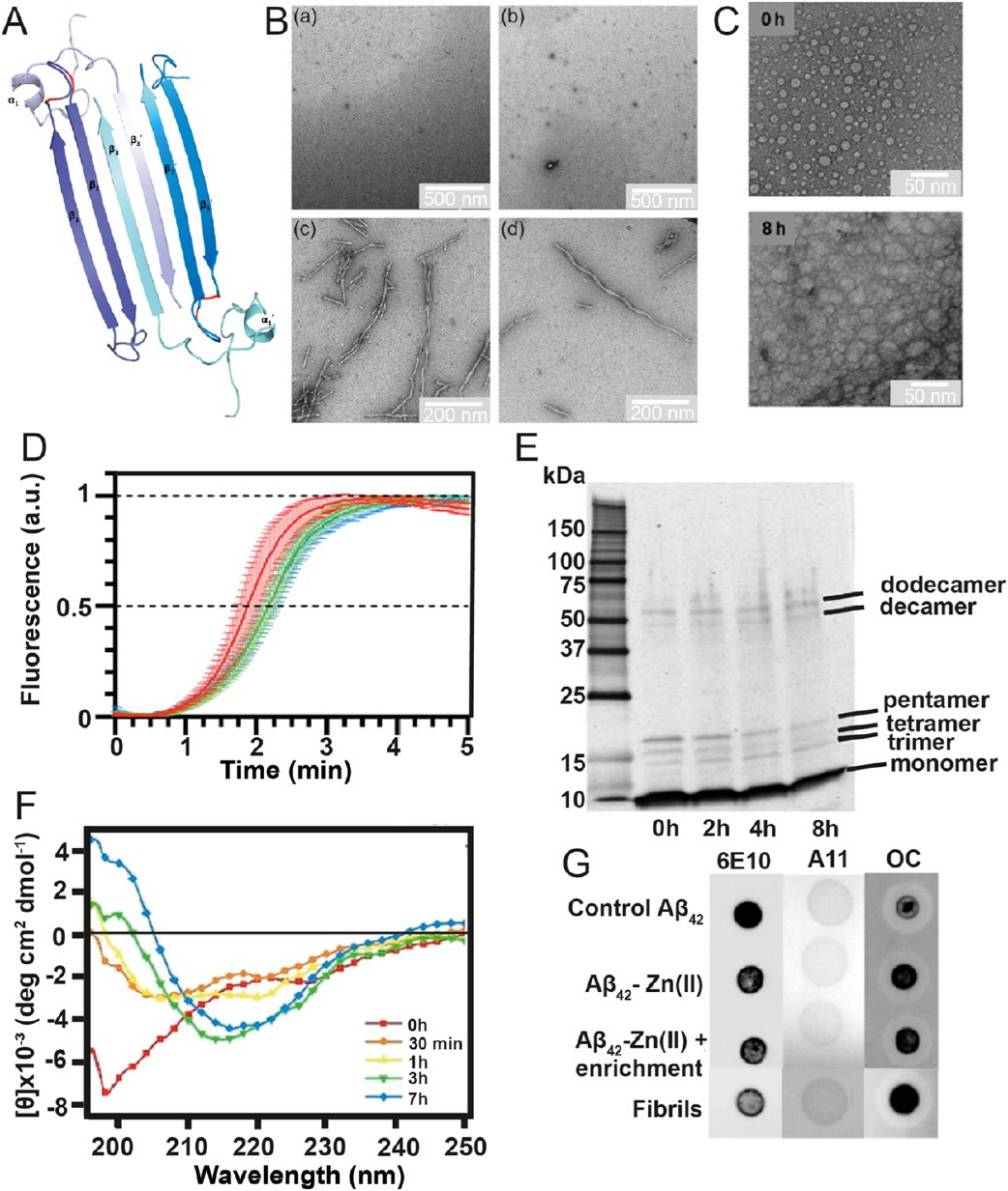
Aβ1–42 Oligomers in vitro. (A) Oligomeric structure of the Aβ1–42 tetramer obtained in a membrane mimicking environment drawn using PyMOL starting from PDB ID 6RHY. Each Aβ1–42 filament was colored differently (Asp23 and Lys28 of the two external segments are in red). (B) NS-EM of 4 μM Aβ1–42 solubilized with 50 mM NaOH and diluted in pH-corrected fibrillization buffer containing 20 mM sodium phosphate, 200 μM EDTA, 1 mM NaN_3_, and 20 μM ThT. (a, b) Representative images of Aβ1–42 samples immediately after the dilution. (c, d) Early growth phase samples from the same experiment, after incubation for ∼1 h in a 96-well microplate at 37 °C.^142^ Reprinted with permission under Creative Commons CC BY 4.0 license from ref 142. Copyright 2022 The Authors. Published by American Chemical Society, licensed under CC BY 4.0. (C) TEM images at the initial and final time points (8 h) of the Aβ1–42 (10 μM) oligomerization in 20 mM phosphate-buffer at pH 7.4 after pretreatment with NH_4_OH 0.16%.^136^ Reprinted in part with permission under Creative Commons CC BY-NC-ND 4.0. License from ref 136. Copyright 2019 The Authors. Published by Wiley-VCH Verlag GmbH & Co. KGaA. (D) Normalized ThT self-assembly kinetics of samples that were subsequently diluted into a preadjusted fibrillization buffer, as described by Taylor et al.^142^ Reprinted in part with permission under Creative Commons CC BY 4.0 license from ref 142. Copyright 2022 The Authors. Published by American Chemical Society. (E) SDS-PAGE results showing the presence of monomeric and different small oligomeric species of the Aβ1–42 oligomerization (10 μM) in 20 mM phosphate-buffer at pH 7.4 after pretreatment with NH_4_OH 0.16%.^136^ Reprinted in part with permission under Creative Commons CC BY-NC-ND 4.0. License from ref 136. Copyright 2019 The Authors. Published by Wiley-VCH Verlag GmbH & Co. KGaA. (F) Secondary structure of the Aβ1–42 oligomerization (25 μM) in 20 mM phosphate-buffer at pH 7.4 after pretreatment with NH_4_OH 0.16% monitored by circular dichroism (CD).^136^ Reprinted in part with permission under Creative Commons CC BY-NC-ND 4.0. License from ref 136. Copyright 2019 The Authors. Published by Wiley-VCH Verlag GmbH & Co. KGaA. (G) Representative dot-blot showing the antibody-specific reactivity of enriched and nonenriched Aβ42-Zn­(II) samples after 20 h of stabilization at 20 °C, together with Aβ42 fibrils. The sequence-specific 6E10 antibody was used as the peptide loading control. A11 and OC were used as conformation-specific antibodies for prefibrillar (A11) and oligomeric (OC) structures.^133^ Reprinted in part with permission under Creative Commons CC-BY 4.0. License from ref 133. Copyright 2024 The Authors. Published by American Chemical Society.

To better understand the native oligomers and their influence on fibril formation, it is crucial to achieve consensus on sample preparation to minimize artifacts. This can be achieved by ensuring the monomerization of the starting peptide sample and performing standardized physical-chemical analysis before any aggregation kinetic assessments. This process should eliminate variability across different batches, which is often used to explain reproducibility issues. Assuming that the end point of the aggregation process is the formation of cross-β-sheet-rich fibrils, a validated aggregation assessment should reproduce the same fibril polymorphism found in amyloid plaques, or at least approach the intermediate nanostructures that lead to this relevant morphology.

We are aware that a strong correlation between the aggregation state of amyloid peptides and their cellular toxicity would provide a better understanding of which aggregate, in terms of size, morphology, and conformation, has the highest toxicity. However, the cell viability assays that have been conducted are generally limited to distinguishing between soluble oligomeric and insoluble fibrillar aggregates without determining the aggregation state at the moment of cellular incubation for toxicity studies. Frequently, the peptides are allowed to aggregate without a common protocol, and at various time points of their aggregation kinetics are incubated with cells, with only a general physicochemical characterization of the heterogeneous solution.^128^ Furthermore, toxicity studies predominantly use undifferentiated neuroblastoma cells, which lack a neuronal phenotype, limiting their utility in studying the link between cytotoxicity and Aβ aggregation states. It is generally recognized that smaller aggregates are more readily internalized by cells, with toxicity mechanisms primarily involving lipid membrane disruption and the activation of inflammatory cascades in microglial cells.^129^ For more details on the toxicity of Aβ aggregates, we refer the reader to the review of Zhao et al.^130^ In our review, we aim to emphasize the importance of a standardized monomerization protocol and highlight how achieving a consensus is essential for future studies to ensure greater consistency in amyloid toxicity research.

In the following sections, a comprehensive overview of various monomerization Aβ protocols used in the literature are presented, along with their impact on the supramolecular structures of the aggregated formed, and, consequently, on the entire aggregation process (Figure 5B–G). The information is listed in Table 2, which includes details of the different protocols and their outcomes in terms of size, morphology, and conformation.

**Table 2:** Preparation Protocols Employed in the Literature to Monomerize the Aβ Peptide during the Aggregation Kinetics Assessment^a^

peptide	pretreatment	oligomers characterization	outcome	refs
**Protocols Based on Guanidinium Hydrochloride (GdnHCl)**				
Aβ1–42	GdnHCl	ThT, AmyloFit	with urea: double filaments, twisted and long; With GdnHCl: short, more than two filaments, less twisted	^131^
Aβ1–42	Gdn.Hcl	ThT	morphology of the fibrils and oligomers was not studied	^132^
Aβ1–42	Gdn.HCl	SDS-PAGE, ThT, ANS, Turbidity, IR, DOT-BLOT, TEM	oligomers ranging in size from 10 to 30 nm, highly hydrophobic, ThT-positive and toxic	^133^
**Protocols Based on Hexafluoroisopropanol (HFIP) or Ammonium Hydroxide (NH_4_OH)**				
Aβ1–42	HFIP (synthetic)	IR, SDS-PAGE, CD	homogeneous Aβ oligomers (β-sheet structures)	^134^
	NaOH (recombinant)			
Aβ1–42	HFIP	ThT, TEM	roundish-like morphology	^135^
Aβ1–42	NH_4_OH	ThT, CD, TEM, SDS-PAGE	species until dodecamers, with a gradual transition from random coil to β-sheet conformation and with globular shape	^136^
Aβ1–42	HFIP	CE, ATR-FTIR, TEM	two main oligomeric populations (low MW oligomers and higher MW species)	^137^
Aβ1–42	HFIP/NH_4_OH	ThT, TEM, DLS, SAXS, SEC	a heterogeneous mixture of cross-β sheets structures of oligomers (80 kDa) and protofibril/early fibril structures	^138^
Aβ1–42	HFIP	ThT, CD, Analytical Ultracentrifugation	11 to 13-mer for the smaller and 17 to 20-mer for the larger oligomers (round in shape)	^139^
Aβ1–42	HFIP	SEC-MALS, DLS, ThT, EM, CD	spherical-globular oligomers, protofibrils with curvilinear structures <200 nm length	^140^
Aβ1–40	HFIP	SDS-PAGE, DLS, CD, ssNMR, TEM	following the fibril formation by TEM according to pH adjustment	^141^
**Protocols Based on Sodium Hydroxide (NaOH)**				
Aβ1–42	NaOH	AF4-MALS, ThT, NS-EM, SEC	β-sheet rich fibrils after 1 h	^142^
Aβ1–40/Aβ1–42	NaOH	ThT, CD, TEM, PICUP	oligomer distribution comprising predominately dimers and trimers, with progressively fewer tetramers; end-stage fibrils with an average diameter of 12 nm and a helical twist with a periodicity of 60–140 nm	^143^
Aβ1–40	NaOH	NMR, ThT, ANS, CD, TEM, AFM, DLS	low abundance oligomers of Aβ without a preferential conformation	^144^

a Abbreviations: Gdn·HCl guanidinium chloride; HFIP hexafluorisopropanol; NaOH sodium hydroxide; NH_4_OH ammonium hydroxide; ThT thioflavin T; IR infrared; SDS-PAGE Sodium Dodecyl Sulfate–PolyAcrylamide Gel Electrophoresis; CD Circular Dichroism; TEM Transmission Electron Microscopy; CE Capillary Electrophoresis; ATR-FTIR attenuated total reflection Fourier transform infrared spectroscopy; DLS Dynamic Light Scattering; SAXS Small-angle X-ray Scattering; SEC Size Exclusion Chromatography; MALS Multi-Angle Light Scattering; EM Electron Microscopy; AF4 Asymmetric Flow Field-Flow Fractionation; NS-EM Negative Staining Electron Microscopy; PICUP Photoinduced cross-linking of unmodified proteins; ANS 8-Anilinonaphthalene-1-sulfonic acid; AFM Atomic Force Microscopy; NMR Nuclear Magnetic Resonance; ssNMR solid-state Nuclear Magnetic Resonance.

##### 2.1.3.1 Protocols Based on Guanidinium Hydrochloride (Gdn·HCl)

Denaturants such as urea and guanidinium hydrochloride (Gdn·HCl) are commonly used to reduce the aggregation rate and stabilize the formation of stable fibrils. By combining experimental methods (e.g., fluorescence spectroscopy) with theoretical approaches (e.g., AmyloFit), it has been shown that denaturants shift the equilibrium toward the unfolded state of each species during the aggregation process. While urea at any concentration primarily affects the nucleation step, particularly secondary nucleation, and does not impact the elongation phase of fibril formation, Gdn·HCl influences the aggregation rate depending on its concentration,^131^ Table 2. Only at concentrations higher than 0.25 M Gdn·HCl stabilizes the unfolded monomeric state, and the denaturing effect dominates. The screening of repulsive electrostatic interactions between peptides by the charged denaturants dominates at lower concentrations, leading to an increased aggregation rate.^131^ This should be considered during the kinetic assessment because denaturants affect the monomers, oligomers, and fibrils, with the nucleation rate being most strongly reduced.

A comparative study where a pretreatment with Gdn·HCl obtained the monomerization of Aβ was recently reported, showing the ability of two solvatochromic probes to detect the early stage aggregative forms of Aβ before being detected by the gold-standard dye, Thioflavin T (ThT).^132^ In this case, an efficient monomerization step was necessary, making it possible to follow the early stages of the entire aggregation process (Table 2). Briefly, the peptide was dissolved in 6 M Gdn·HCl and incubated for 5 min. Aβ solutions were centrifuged at 16000 rpm for 15 min at 4 °C and injected into a size exclusion column chromatography (SEC). This pretreatment showed that the intensity emitted by these fluorophores increased earlier than that of ThT, proving to be suitable for monitoring the early phases of the amyloid self-assembly.

Previous observations regarding the ability of Aβ to interact with metal ions led researchers to develop a standardized method for preparing stable and homogeneous oligomeric structures.^133^ This protocol involved a monomerization step, followed by stabilization with Zn­(II), and then the isolation of the generated oligomers. Aβ was initially dissolved in 6 M Gdn·HCl in 50 mM ammonium acetate buffer at pH 8.5 and kept on ice for at least 1 h. Monomers were separated from aggregates using SEC, followed by a second monomerization protocol. For the second monomerization step, the peptide was solubilized in HFIP, sonicated for 10 min at room temperature, and then incubated overnight at 4 °C. After evaporating the organic solvent, the peptide was redissolved in DMSO, spun down, sonicated again for 10 min, and centrifuged at 13,000 rpm for 3 min at 20 °C. The supernatant was diluted in buffer to initiate aggregation, and ZnCl_2_ was added at a 1:10 molar ratio. The mixture was incubated at 20 °C for 20 h. After incubation, the sample was centrifuged at 13,000 rpm for 30 min at 20 °C, and the pellet fractions were resuspended in 20 mM Tris-HCl buffer (pH 7.4) containing 60 μM ZnCl_2_. This protocol generated oligomers ranging in size from 10 to 30 nm, which were highly hydrophobic, ThT-positive, and toxic to neuroblastoma cells^133^ (Table 2).

##### 2.1.3.2 Protocols Based on Hexafluoroisopropanol (HFIP) or Ammonium Hydroxide (NH_4_OH)

Infrared spectroscopy has proven to be a valuable method for characterizing both homogeneous and heterogeneous Aβ oligomer preparations. It has been shown that the spectral position and bandwidth of the Amide I band, associated with β-sheets, can be used to analyze the secondary structure, size, and distribution of aggregates. For the preparation of stable oligomeric solutions, synthetic Aβ peptides are typically pretreated with hexafluoroisopropanol (HFIP), while recombinant peptides are treated with ammonium hydroxide (NH_4_OH) to facilitate the monomerization step required to achieve stable and high-quality oligomer preparations (Table 2). In this protocol, Aβ is solubilized in dimethyl sulfoxide (DMSO) at a concentration of 4 mg/mL, passed through a HiTrap desalting column, and eluted with 10 mM NaOH (pH 12.0–12.3). The monomeric fractions are then frozen and stored at −80 °C until needed. To stabilize the oligomer solutions, a detergent (0.05–0.2% SDS) is added to 80–100 μM of peptide in PBS (20 mM NaH_2_PO_4_, 140 mM NaCl, pH 7.4), and the mixture is incubated at 37 °C for 24 h. After incubation, the solution is flash-frozen and stored at −20 °C.^134^ This protocol results in homogeneous Aβ oligomers with well-defined β-sheet structures, as confirmed by IR spectroscopy. Transmission electron microscopy (TEM) was used to qualitatively assess the presence or absence of fibrils immediately after postpeptide resolubilization and upon completion of the kinetics, without detailed morphological characterization (Table 2).

Fluorescence spectroscopy is widely used to follow the self-assembly of amyloid proteins, particularly by employing solvatochromic fluorescent probes that sense hydrophobic surfaces. Mainly, it is utilized to follow the formation of early stage species that ThT cannot detect. Two fluorophores have recently been reported to bind to early stage aggregates of Aβ: aminonaphthalene 2-cyanoacrylate-spiropyran (AN-SP)^135^ and triazole-containing boron-dipyrromethene (taBODIPY).^68^ Both molecules increase their fluorescence intensity in the presence of early stage Aβ aggregates, respectively. In the case of AN-SP,^67^ the Aβ was dissolved in HFIP for 10–20 min at room temperature to reduce the aggregation rate and focus on the early stage oligomers. The resulting seedless Aβ solution was mixed with water for 10–20 min at room temperature. Then the samples were centrifuged for 15 min at 14,000 rpm and the supernatant fraction (pH 2.8–3.5) was subjected to a gentle stream of N_2_ for 5–10 min to evaporate the HFIP. The samples were stirred again at 500 rpm using a Teflon-coated micro stir bar for 24–48 h at 22 °C. A further characterization of the sample was performed using TEM, showing a roundish-like morphology. In the case of taBODIPY,^68^ Aβ was dissolved in 0.16% NH_4_OH to remove preformed aggregates for 15–20 min at room temperature, followed by lyophilization. The peptide was then reconstituted in 1% NH_4_OH with sonication at 25 °C for 1 min before being dissolved in a 20 mM phosphate buffer at pH 7.4 to provide the appropriate concentration for the analysis. The characterization by different physicochemical techniques, such as TEM (Figure 5C), SDS-PAGE (Figure 5E), and circular dichroism (CD) (Figure 5F), allowed a thorough characterization of the sample, demonstrating the presence of species until dodecamers, with a gradual transition from random coil to β-sheet conformation and globular shape^136^ (Table 2).

Capillary electrophoresis (CE) has been employed as a separation method to study the effect of different Aβ sample preparation protocols on forming the assemblies in solution. Three main reconstitution protocols were assessed, all using HFIP (221 μM at 4 °C for 30 min or 149 μM at room temperature overnight) to monomerize the peptide. After the evaporation of HFIP, Aβ was successively redissolved in 20 mM phosphate buffer (pH 7.4) without increasing the concentration of the organic solvent (DMSO or a mixture of acetonitrile), followed by the addition of 300 μM Na_2_CO_3_ and 250 mM NaOH (48.3:48.3:3.4, v/v/v) with subsequent dilution in 20 mM phosphate buffer (pH 7.4). Despite the CE separation being limited to two main oligomeric populations (low MW oligomers and higher MW species), it was clear from the electropherogram that, immediately after the redissolution of the dried film following HFIP evaporation, the earlier migrating peak corresponding to the monomer was the most abundant. However, aggregation had already begun, as two additional peaks were present. TEM images taken after immediate resolubilization revealed that fibrils were also present in this protocol, confirming that HFIP is unsuitable for completely monomerizing the peptide,^137^ Table 2. If complete monomerization is not achieved, residual preformed aggregates remain and act as seeds, accelerating the aggregation of monomers. In such cases, the experimental setup does not fully represent the intrinsic monomer misfolding mechanism driving the aggregation process. This may distort aggregation kinetics and bypass the critical conformational transition from the physiological to the pathological monomeric state. The addition of DMSO did not affect oligomerization but allowed the oligomers to remain soluble, thereby ensuring greater stability of the oligomer samples. Finally, the inclusion of NaOH in the buffer reduced the aggregation tendency of the peptide.^137^

The pretreatments with HFIP or NH_4_OH were compared in a recent study to assess how to obtain a more homogeneous solution, primarily through strong alkali preparation protocols. The effect of the treatment was investigated by combining ThT fluorescence spectroscopy, TEM, dynamic light scattering (DLS), SEC, and small-angle X-ray scattering (SAXS).^138^ The peptide was prepared by a previous incubation in 100% HFIP (1 mg/mL) followed by 5 min of sonication and drying under nitrogen steam or 10% (w/v) NH_4_OH (0.5 mg/mL) followed by 5 min of sonication and lyophilization. Immediately before use, the pretreated samples were then dissolved in 60 mM NaOH and successively diluted in PBS (136.89 mM NaCl, 2.68 mM KCl, 6.39 mM Na_2_PO_4_, and 1.47 mM KH_2_PO_4_) to achieve a final pH of 7.4. Aggregation was monitored at 37 °C with agitation every 7 min. The use of HFIP resulted in a heterogeneous starting peptide self-association state, which affected the self-association kinetics by increasing the fibril formation rate. It is important to note that an increase in ThT fluorescence intensity (approximately 10 arbitrary units) over the first hour was observed, likely due to the concurrent presence of cross-β sheet structures from preformed oligomers or small aggregates (Table 2). Through DLS, HFIP-treated samples showed high variability in size (3–100 nm), with a radius of 8 nm and 50% polydispersity. Conversely, NH_4_OH-treated samples were more consistent, with radii ranging from 1–10 nm and 30% polydispersity, averaging 3.5 nm. Size exclusion analysis confirmed these findings, revealing that HFIP treatment resulted in a heterogeneous mix of species with large oligomers (MW greater than 80 kDa). These oligomers were less structured and characterized by SAXS analysis. NH4OH-treated Aβ42 exhibited a more defined bimodal distribution, with ∼ 90 and ∼ 10% monomers and medium-sized oligomers (∼30–50 kDa), respectively.

TEM micrographs showed the presence of protofibril/early fibril structures in samples pretreated with HFIP, more so than in those treated with NH_4_OH. Pretreatment methods were found to be important in determining Aβ toxicity in PC-12 cells. In both cases, toxicity was observed 72–96 h post-treatment, with no toxicity observed at an earlier time point (48 h).^138^ This could be explained by the fact that high molecular weight oligomers formed under these protocols were not immediately toxic to PC-12 culture cells. Thus, the absence of efficient monomerization and the simultaneous presence of early aggregates could affect the kinetic pathway, leading to a nontoxic aggregation process.

Analytical ultracentrifugation, particularly sedimentation velocity (SV) centrifugation, has also been used to study freshly prepared solutions of Aβ to gain insights into its early self-assembly processes during the lag phase of amyloid formation.^139^ SV analysis at pH 10 confirmed that the Aβ solution consisted of 95% monomers after overnight pretreatment at room temperature in HFIP. The peptide was then dissolved in a 10 mM phosphate buffer at pH 7.4 to initiate aggregation. Approximately 6% of aggregates were detected, consisting either of residually undissolved material after HFIP pretreatment or newly formed assemblies after dissolution. The study allowed for the identification of distinct oligomeric species in solution during the lag phase. These oligomers appeared to be globular in shape and always sedimented alongside smaller species, thus maintaining equilibrium between distinct oligomeric species and smaller oligomers and monomers. The two recurring species were interpreted as an 11- to 13-mer for the smallest oligomers and a 17 to 20-mer for the largest oligomers, (Table 2). The number of monomers for each oligomeric species supports a model of self-assembly based on a hexameric building block.^139^

Nichols et al. compared two *in vitro* Aβ aggregate preparations for biological studies, specifically forming protofibrils or oligomers.^140^ They employed an SEC isolation coupled to multiangle light-scattering analysis (SEC-MALS), dye binding, and microscopy to characterize the biophysical features, particularly focusing on molecular weight and root-mean-square radius. The monomerization of the peptide was conducted in HFIP with slow evaporation overnight at room temperature in a fume hood. To remove any residual HFIP, the samples were successively vacuum-centrifuged. To prepare the protofibrils, Aβ was dissolved in 50 mM NaOH before dilution in the buffer, while for oligomer preparation, DMSO was used. The authors emphasized that Aβ must be pretreated under alkaline conditions to avoid crossing the Aβ isoelectric point (pI 5.5) when transferring a direct aqueous solution of the peptide into buffers, which could lead to a significant decrease in polydispersity. Surprisingly, SEC-MALS analyses showed that oligomers were larger in multiple preparations than protofibrils. The two species exhibited similarities in ThT binding, fluorescence, and extended conformation. However, a notable difference in morphology was observed. While oligomers were spherical-globular, Aβ protofibrils exhibited curvilinear structures less than 200 nm in length. Both protofibrils and oligomers may coexist and share size and structural similarities (Table 2); the key distinction between them lies in the solvent-exposed surface of the aggregate.^140^

The characterization of successive stages of amyloid-β self-assembly has also been reported through NMR analysis combined with dynamic nuclear polarization.^141^ The abundance and distribution of aggregated species relative to monomers in the sample were determined using four independent techniques: Photoinduced cross-linking of unmodified proteins (PICUP), DLS, TEM, and sedimentation velocity. To achieve this, HFIP-treated peptide (2 mg/mL, room temperature, 30 min to 1 h) was dissolved at a 2.5 mM concentration in 20 mM NaOH, and pH 12. At this high pH, the specimens showed a nearly clear TEM image, with low coverage of amorphous aggregates that may have developed during drying on the grid or due to ineffective disaggregation by the HFIP treatment. Lowering the pH by adding 250 mM phosphate buffer (pH 7.4) with a small quantity of concentrated HCl to adjust to neutral pH resulted in a high density of irregularly shaped assemblies within 2 min after pH adjustment. Incubation at 24 °C for 4–5 h led to high-density, short, and curved protofibrils, as confirmed by ThT experiments. However, the plateau was reached after approximately 10 h, corresponding to 40% of the fluorescence level observed after conversion to mature fibrils. Fibrils were obtained by sonication of the protofibril solution and further incubation at 24 °C for another 12 h (Table 2). TEM images showed a mixture of protofibrils and fibrils, which were thicker, longer, and less curved than the sample before sonication. Only after 3–4 repetitions of the sonication/incubation procedure could complete conversion to mature fibrils be achieved.^141^

##### 2.1.3.3 Protocols Based on Sodium Hydroxide (NaOH)

By employing a novel fractionation method called asymmetric flow field-flow fractionation (AF4), Taylor et al. demonstrated that the commonly recommended dilute base treatments for resolubilization, typically resulting in a pH < 11, are insufficient to fully monomerize the peptide or to prevent seeding and off-pathway aggregation. Instead, they suggested using a more concentrated base (50 mM NaOH, resulting in a pH of 12.5), coupled with sonication, to fully solubilize the peptide and ensure that the samples are free from fibril seeds and off-pathway aggregates. They combined this new method with ThT fluorescence spectroscopy, negative-stain TEM, and SEC, demonstrating that the widely used Aβ solubilization protocols resulted in unreliable self-assembly kinetics due to incomplete monomerization (Figure 5D). In contrast, Aβ samples solubilized in 50 mM NaOH exhibited highly reproducible, unseeded self-assembly kinetics, leading to proper folding into β-sheet fibrils. Fibrils were observed after at least 1 h of incubation under fibrillization conditions with a pH-corrected fibrillization buffer (Figure 5B)^142^ (Table 2).

The use of NaOH to prepare Aβ samples before self-assembly assessment was also employed in a research article aimed at identifying the key regions and residues controlling Aβ folding. The peptide was initially dissolved in a mixture of 10% (v/v) 60 mM NaOH, 45% (v/v) water, and 45% (v/v) 22.2 mM sodium phosphate, pH 7.4, on ice. After sonication for 1 min and filtration using a 30 kDa molecular mass cutoff, the peptide concentration was determined by tyrosine absorption at 276 nm and adjusted to 40 μM using 10 mM sodium phosphate, pH 7.4, prior to analysis through cross-linking and SDS-PAGE, ThT fluorescence, CD, and TEM.^143^

PICUP revealed that Aβ produced an oligomer distribution predominantly consisting of dimers and trimers, with progressively fewer tetramers and pentamers. ThT spectroscopy and TEM analysis confirmed that these oligomers could form end-stage fibrils with an average diameter of 12 nm and a helical twist with a 60–140 nm periodicity (Table 2). Tyr–Tyr cross-linking has emerged as a valuable tool for understanding the oligomerization dynamics of Aβ. This covalent modification, driven by oxidative processes, facilitates the stabilization of Aβ oligomers and offers insights into their assembly mechanisms. Notably, the PICUP technique has been employed to elucidate these dynamics, offering a mechanistic perspective on Aβ aggregation.^145^ Recent studies have detected Tyr-Tyr cross-links in both Aβ and tau deposits, the primary constituents of amyloid plaques and neurofibrillary tangles in AD, respectively.^146^ However, as highlighted in the comprehensive review by Maina et al.,^147^ there is no consensus regarding the impact of Tyr–Tyr cross-linking on Aβ’s function, aggregation propensity, and toxicity. For an in-depth exploration of this topic, readers are encouraged to consult the aforementioned review.^147^

A similar approach was employed to characterize low-abundance oligomers of Aβ using high-resolution NMR analysis in combination with other biophysical experiments, including CD and fluorescence spectroscopy (ThT, bis-ANR), DLS, AFM, and TEM (Figure 6)^144^ (Table 2). Again, a pretreatment with a basic solution was demonstrated to be a valuable method for preparing the peptide to control the aggregation kinetics, starting from a monomeric sample and obtaining the coexistence of early soluble and mature insoluble aggregates. Thus, by controlling the pH of the reconstitution buffer, it is possible to manage the state of aggregation of Aβ.

**Figure 6:**
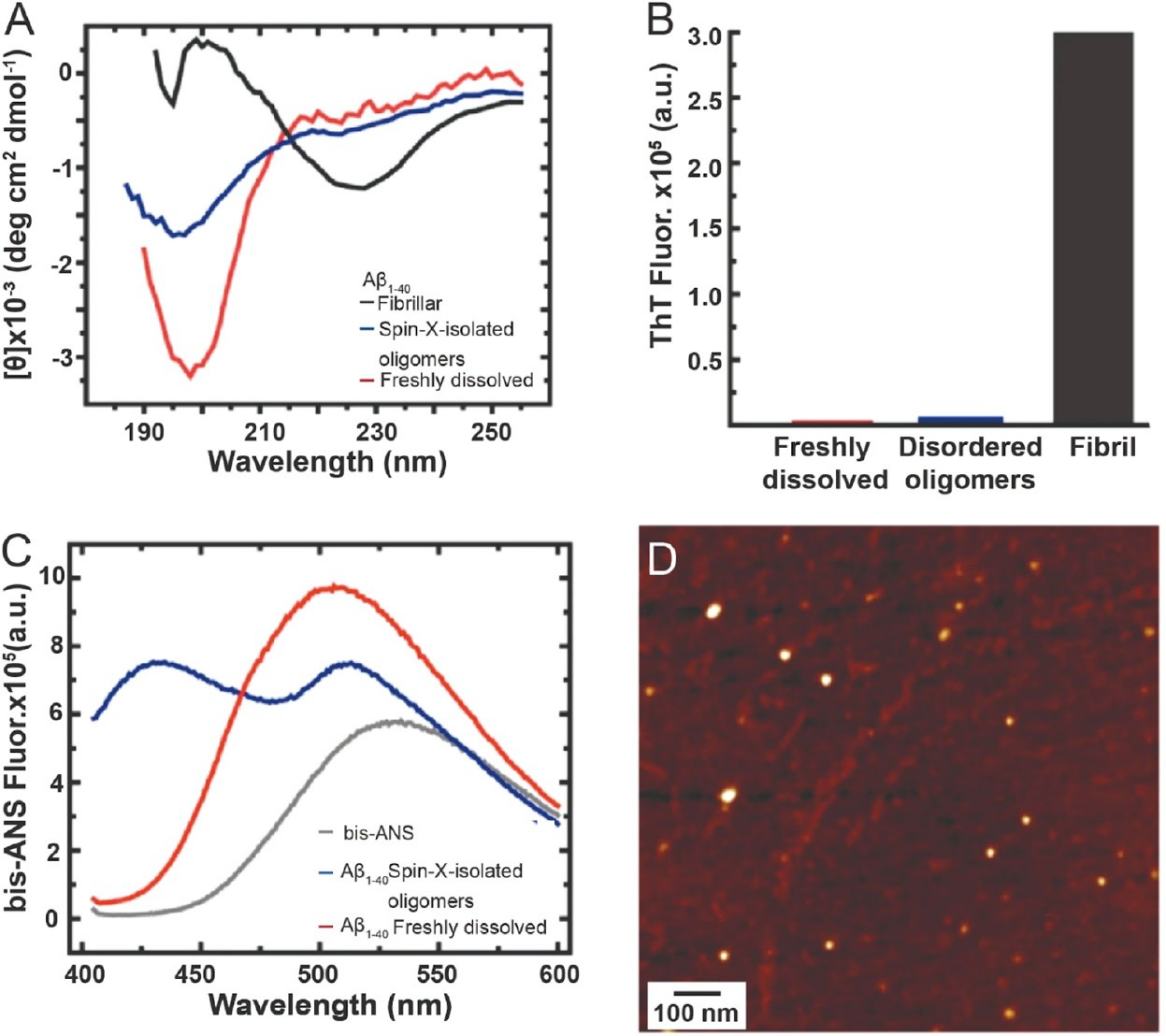
Biophysical characterization of Aβ1–40 disordered oligomers as reported by Kotler et al. (A) CD spectra, (B) ThT fluorescence, and (C) bis-ANS fluorescence of fibrillar (black), spin-X-isolated oligomers (blue), and freshly dissolved (red) samples of Aβ1–40. The emission spectrum of bis-ANS in solution is shown in gray in panel C. (D) Representative AFM image of the Aβ1–40 oligomers. All experiments were performed in a 10 mM sodium phosphate buffer, pH 7.4, at 25 °C.^144^ Reprinted in part with the permission of Creative Commons CC BY license from ref 144. Copyright 2015 The Authors. Published by Springer Nature.

#### 2.1.4 Key Takeaways on the Aggregation of Aβ Peptides

Despite all the relevant work, it remains challenging to select the optimal treatment for Aβ peptide to achieve the suitable monomer conformation that correlates with a specific aggregation process, leading to pathologically relevant fibrils. A consensus is needed for a universally accepted protocol to monomerize the Aβ peptide as effectively and reproducibly as possible. HFIP seems preferentially used to disassemble previously existing aggregates, but pretreatment at basic pH with NaOH appears to ensure complete monomerization to initiate the *in vitro* assessment.

Based on these details, we outline key considerations and recommendations to guide the selection of an appropriate monomerization protocol.

- 6 M GndHCl: This treatment is effective for monomerization; however, it needs subsequent steps, such as SEC, to ensure a sample predominantly composed of monomeric species.
- HFIP and NH_4_OH: These methods often require additional processing, either through a combination of solvents or steps involving varying aqueous concentrations of NH_4_OH. These treatments may not fully guarantee monomerization, as residual aggregates can catalyze aggregation toward amorphous, insoluble species.
- 60 mM NaOH: Before any analysis, maintaining an extremely basic pH by treating the peptide with 60 mM NaOH has been shown to monomerize the peptides, ensuring reproducible aggregation kinetics effectively.

Nevertheless, characterization of the final peptide sample must be performed through a multimodal approach, with complementary methods implemented to better assess the reproducibility and quality of the aggregates and the reliability of the samples.^148,149^ Phosphate buffer at low concentrations remains the preferred choice for studying aggregation, as the increased apparent dielectric constant of water due to the salts can promote necessary protein–protein interactions, and phosphate ions do not interfere with spectroscopic methods. A combination of complementary physical-chemical methods is widely accepted as a valuable approach to verifying the quality of the amyloid sample, with the reproducibility of ThT kinetics serving as an easy way to compare treatments.

Furthermore, systematic TEM analysis should be performed to verify that the ThT-positive aggregates exhibit fibril morphology similar to those found in patients’ brains. Finally, it was recently reported that mesoscopic polymorphisms of amyloids can also be influenced by the possibility of a single peptide folding into both irreversible and reversible amyloids, each presumably having distinct amyloid motifs despite an unchanged primary sequence. A peptide is typically considered to adopt a single lowest-energy state, however, this is not the case for intrinsically disordered peptides. Moreover, peptides undergoing misfolding can adopt either a canonical cross-β-sheet structure (irreversible amyloid state) or a less-ordered morphology (reversible amyloid state).^150^ While a canonical cross-β-sheet structure is observed only for irreversible amyloids, a less-ordered coexistence of β-sheet and helical secondary structures, originating from a partially unfolded state, is found for the reversible amyloids, as if the peptide retains a “memory” of the original folded protein precursor. To support this observation, the authors combined AFM, cryo-EM, and solid-state NMR, concluding that a complete unfolding of the native protein precursor is required to establish irreversible amyloid fibrils.^150^

### 2.2 CeD and Proteolytically Resistant Gliadin Peptide Nanostructures

#### 2.2.1 Peptide and Protein Aggregates in CeD: Expanding the Theoretical Framework

Gliadins are proteins in wheat gluten^151^ relevant to the Western daily diet that are found mainly in bread, pasta, and cookies. Moreover, wheat gluten is also a principal component of veggie-ready food because of its meat-like texture in seitan and Yuba.^152–155^ Nevertheless, gliadins, especially the isoforms α-2, 9, and γ, have been identified as the primary source of toxic and immunoreactive peptides that are resistant to proteolysis.^156,157^ Some gluten immunogenic peptides (GIPs) can also reach blood circulation,^158–161^ being excreted in the stool and urine when gluten is in the diet,^38,40,160^ highlighting their resistance to proteolysis *in vivo* and, importantly, their systemic distribution. Some of the PRGPs can trigger a wide variety of diseases GRDs in predisposed people, and they are also connected with type-I diabetes.^34,162–164^ Among GRDs, CeD is an autoimmune enteropathy triggered by the consumption of gluten from wheat, barley, rye, and some varieties of oats.^52^ CeD is characterized by inflammation, increased gut permeability, and severe damage in the small intestine.^165^ A genetic predisposition, such as the presence of human leucocyte antigen (HLA)-DQ2 and HLA-DQ8 molecules, has been recognized in most patients with CeD.^165,166^ However, the abundance of 30–40% of these molecules in the total population does not explain the average worldwide prevalence of 1%.^167–170^ Moreover, other GRDs, such as dermatitis herpetiform, gluten ataxia, or, more recently, nonceliac gluten (wheat) sensitivity, have been described as extra-intestinal manifestations in some gluten-sensitive individuals where other organs are affected.^171^ Despite different research efforts, the only treatment for these disorders is a gluten-free diet.^172^

Shan et al. showed by a comprehensive study of the enzymatic digestion of α-2 gliadin, showing that a large 33-mer peptide remained unchanged, not only *in vitro* but also when exposed to human small intestine biopsies and in perfusion experiments in mouse models.^173^ The 33-mer gliadin peptide, (^57^LQLQPF­(PQP**Q**LPY)_3_PQPQPF^89^) demonstrated high-affinity binding to tissue transglutaminase-2 (tTG2), the CeD autoantigen. tTG2 plays a central role in CeD onset by promoting two key reactions: self-cross-linking with specific gliadin peptides^174–176^ and the selective deamidation of glutamine residues, converting them into glutamic acid residues.^177,178^ Specifically, the reaction between the 33-mer gliadin peptide and tTG2 produced a 33-mer deamidated gliadin peptide (33-mer DGP, ^57^LQLQPF­(PQP**E**LPY)­3PQPQPF^89^).^177^ The deamidated sequence is preferentially presented by the HLA-DQ2/8 complex in most of the patients with CeD patients.^178–180^ Later, it was demonstrated that 33-mer DGP accumulates and forms aggregates in mouse models.^162^ Another widely studied PRGP is the p31–43 peptide (^31^LGQQQPFPQQPQQPFPQ^43^),^181–183^ which forms oligomers and promotes direct damage to the small intestine and triggers an innate immune response in mouse models, suggesting its toxic nature.^51,184^ Interestingly, this peptide activates the tTG2 but not its substrate.^183,185^

The formation of aggregates in connection with CeD is not limited to these two PRGPs. Recently, it was shown that TG2:33-mer immunocomplexes and other tTG2-gluten multimers promote a strong immune response, which not only activates T-cells but also triggers B-cell activation,^186^ highlighting a pathological role of the deposits found in the gut biopsies of patients with CeD.^187,188^ In this context, anti-TG2 IgA deposits have been detected in early intestinal biopsies from infants at risk of CeD, demonstrating predictive value for developing villous atrophy.^188,189^ Similarly, anti-TG6 IgA deposits have been identified in cerebellar blood vessels in patients with gluten ataxia, with these deposits disappearing upon adherence to a gluten-free diet.^190,191^ In dermatitis herpetiformis, a skin manifestation of CeD, anti-tTG3 complexes had been described as deposits in the epidermis.^192,193^

Although GRDs, particularly CeD, involve PRGPs and protein aggregates with positive anti-tTG2, anti-tTG3, or anti-TG6 signals in their pathomechanism, they are not yet classified as protein misfolding disorders. Nevertheless, Bethune and Khosla previously proposed that PRGPs behave like nonreplicating pathogens, primarily due to their activation of innate and humoral immunity.^194^ Recently, it was reported that some pathogenic PRGPs sequences share structural similarity^195^ with within pathogen-derived proteins, e.g., extracellular proteins from Streptococcus pneumoniae, *Granulicatella sp* and more than 100 proteins from enteric coccidian parasites proteins. Beyond their structural features at the amino acid level, experimental evidence shows that PRGPs form oligomers and multimers with TG in different organs. Expanding the theoretical framework to include the aggregation capabilities of PRGPs and their immunocomplexes could open the door to developing effective strategies to treat or prevent CeD and potentially other GRDs. While further studies are essential to elucidate the role of these exogenous peptides and their immunocomplexes in humans before the onset of disease, their accumulation and aggregation may serve as early triggers in GRDs, similar to what is known for protein misfolding pathologies such as AD.

#### 2.2.2 Nanostructures of PRGPs: Toward Understanding the Early Stages of CeD

Considering the relevance of the 33-mer gliadin peptide in CeD, in 2014, some of us further investigated its structural behavior, discovering that the 33-mer undergoes a structural transition and forms oligomers and protofilaments under physiologically relevant conditions, similar to Aβ peptides.^36^ After this report, we and others have focused on detailing the aggregation mechanism of 33-mer gliadin peptides, p31–43, and other mixtures of PRGPs obtained after enzymatic digestion by human enzymes, connecting them with biological and pathological processes using cellular models. A review comparing the two different 33-mer gliadin peptide oligomers and their cellular effect was recently published.^26^ Here, we first describe their structural features in comparison with the less studied PRGPs. Therefore, the protocols for forming different nanostructures and the main characterization methods used to confirm the nanostructures will be presented in connection with their cellular effects in the following subsections and are listed in Table 3. Moreover, a schematic representation of immune and cellular pathways triggered by PRGPs and their nanostructures in the gut and epithelial models is shown in Figure 7.

**Figure 7:**
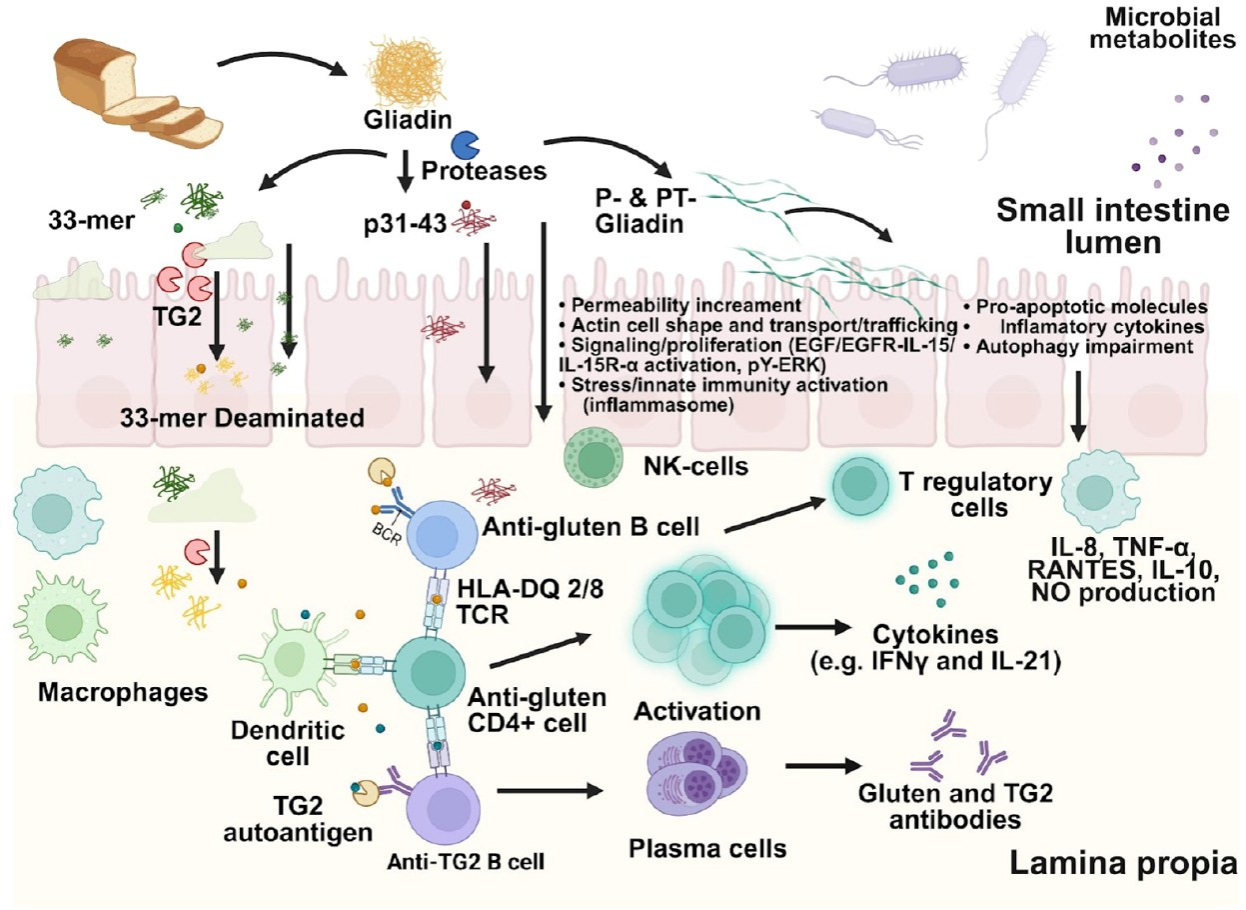
Schematic representation of the known effects of PRPGs on the gut and the activation of the immune response. Gliadin is incomplete degraded, producing peptides that can cross the gut mucosa. The 33-mer, in particular, can traverse the gut mucosa and has been shown in cellular models that 33-mer large superstructures can activate macrophages, inducing an innate response. Additionally, the 33.mer and its deamidated version (33-mer Deamidated, 33-mer DGP) produced by the TG2 can promote an adaptive immune response, leading to the production of specific antibodies and T cells. On the contrary, the effects of the p31–43 peptide are associated with the gut mucosa and induction of innate responses. P- and PT-gliadin peptides have been shown to promote inflammatory molecules and impair autophagy in epithelial models. Only those peptides that form nanostructures are shown. Created with BioRender.com.

**Table 3:** Nanostructures of PRGPs: Preparation, Characterization *In Vitro* and Their Cellular Effect^a^

peptide	sample preparation	characterization	secondary structure	nanostructures	cellular effects	refs
33-mer	Stock solution (3 mg/mL) in Milli-Q water or/PBS or/citric acid buffer. Followed by short vortex/sonication. After 2 days at 4 °C, the system reaches equilibrium. Storage at –20 °C. Syringe filtration at high concentrations to obtain smaller oligomers. Pretreatment with HFIP did not show changes.	*Oligomerization Pathway*: CD, ATR-FTIR, steady-state and time-resolved intrinsic tyrosine fluorescence spectroscopy, negative for ThT assay. DLS and surface tension (Plate method according to Wilhelmy)	PPII-helix and β-parallel structures depending on peptide concentration	oligomers, protofilaments, and thin plate structures	Production of IP-10 and TNF−α via TLR2/4 receptors in macrophage cell line.	^36,37,50,196^
		*Microscopy*: NS-EM, SEM, AFM, HIM, Cryo-EM				
33-mer DGP	Stock solution (3 mg/mL) in Milli-Q water or PBS, 4 °C for 1 day. Followed by short vortex/sonication. Storage at –20 °C	*Oligomerization Pathway*: CD, steady-state fluorescence anisotropy, surface tension (Plate method according to Wilhelmy)	major PPII-helix and minor β structures	spherical oligomers	Increased Permeability and ZO-1 Remodeling in Caco-2 Cell Model	^197^
		*Microscopy*: NS-EM, AFM				
p31–43	Stock solution in Milli-Q water (1 to 3 mg/mL) and stored at –20 °C	*Oligomerization Pathway:* CD, DLS, steady-state fluorescence spectroscopy, steady-state fluorescence anisotropy	major PPII-helix and minor β- structures	spherical oligomers and amorphous aggregates	Disruption of the gut barrier induces enteropathy in mice and promotes inflammatory responses via NLP3 inflammasome.	^51,198,199^
	NMR studies H_2_O/D_2_O 90:10 (v/v) mixture and with SDS at pH 4.6 ± 0.1	*By NMR:* Possible conformational equilibrium between monomers and nanostructures				
		*Microscopy*: NS-EM and AFM				
P- gliadin	Stock solution at (1 mg/mL) at pH 3.0, stabilization at 4 °C overnight, followed by centrifugation. Supernatant as the working solution (0.1 mg/mL). Reach 37 °C and addition of Pepsin. Reaction for 2 h. Inhibition of the enzyme at 95 °C (10 min).	*Oligomerization Pathway*: SDS-PAGE. UV–vis spectroscopy, DLS, CD, fluorescence spectroscopy (intrinsic tryptophan fluorescence and oligomers/amyloid sensitive probes: NR, BODIPY, and ThT)	PPII-helix, β- structures, and minor α-helix.	oligomers and amyloid-like fibrils	Induction of inflammation and pro-apoptotic genes and the CXCL-8/CXCR3 axis in Caco-2 cell model.	^196^
		*Microscopy*: NS-EM				
T-gliadin	Gliadin was suspended in deionized H_2_O (2.5 wt %), and the solution was taken to pH 8.0 by NaOH (1 M). Trypsin digestion occurs at 300 rpm at 37 °C, with NaOH additions to maintain the pH. The protein hydrolysates were heated to 95 °C for 15 min to deactivate the enzyme. Next, the solutions were cooled to room temperature and centrifuged at 18,600*g* for 15 min. The supernatant was lyophilized and resuspended in water.	*Aggregation Pathway*: Fluorescence spectroscopy (ThT), DLS, ζ potential measurements, FTIR	β-structures	amyloid-like fibrils	-	^200,201^
		*Microscopy*: NS-EM				
PT-gliadin	Digestion of gliadin (1 g/mL, 500 mL) with pepsin (1 g) in 0.2 N HCl at 37 °C for 2 h followed by pH neutralization (2 N NaOH; pH 7.4) followed by proteolysis with trypsin (1 g) at 37 °C for 4 h. Finally, the mixture was boiled to inactivate enzymes for 30 min and was stored at –20 °C.	*Peptide sequence and molecular weight analysis:* SDS-PAGE, DLSHPLC and MS	-	fibrils and amorphous aggregates	Uptake, reduces cell viability, and impairs autophagy in Caco-2 cells.	^202,203^

	In several preparations, BBM or/and elastase/carboxypeptidase A were added.	*Microscopy*: NS-EM and fluorescent microscopy				
PT-gliadin cationic	Gliadin (30 g) was suspended in 400 mL of deionized water (pH 2.0) and digested by pepsin (2000 U/mL) for 2 h at 37 °C. pH was neutralized to 7 with 2 M NaOH. Then, the suspension was further digested by trypsin (100 U/mL) for 2 h at 37 °C. The reaction was stopped by boiling for 15 min, folllowed by centrifugation at 7000*g* for 20 min at 4 °C. After supernatant collection, a second centrifugation at 200,000*g* for 30 min at 4 °C was performed. The precipitate was collected, resuspended in deionized water, and centrifuged at 200,000*g* for 30 min at 4 °C. The pellet was the working solution.	*Peptide sequence and molecular weight analysis:* SDS-PAGE, HPLC, and MS	PPII-helix ( <0.1 mg/mL); compacted β-sheet structure (>0.3 mg/mL)	oligomers/nanoparticles	Penetrates mucus in HT29-MTX/Caco-2 cells coculture. Inhibits mucus production.	^204^
		*Oligomerization Pathway*: CD, DLS, fluorescence spectroscopy (ThT)				
		*Microscopy*: NS-EM				

a Abbreviations: ThT thioflavin T; FT-IR Fourier Transform I Spectroscopy infrared; ATR-IR Attenuated Total Reflectance Infrared Spectroscopy; SDS Sodium Dodecyl Sulfate, SDS-PAGE Sodium Dodecyl Sulfate - PolyAcrylamide Gel Electrophoresis; CD Circular Dichroism; NR nile red; NS-EM Negative Staining Electron Microscopy; HIM Helium Ion Microscopy; DLS Dynamic Light Scattering; SAXS Small-angle X-ray Scattering; AFM Atomic Force Microscopy; SEM Scanning Electron Microscopy; Cryo-EM Cryo-electron Microscopy; NMR Nuclear Magnetic Resonance; HPLC High-Performance Liquid Chromatography; MS Mass spectrometry; ZO-1 zonula occludens 1.

##### 2.2.2.1 Nanostructures of 33-Mer Gliadin Peptides

After the discovery of the 33-mer gliadin peptide,^173,205^ its intrinsic structural features explaining toxicity and immunoreactivity were initially thought to be limited by its high content of proline residues and the PPII-helix motif. The PPII-helix is a flexible left-handed helix in equilibrium with other conformations, mainly β-type structures, turns, and β-sheets, all of which have self-assembly capabilities. This equilibrium could shift depending on temperature, with the PPII-helix being predominant at lower temperatures.^206,207^ The PPII-helix has recently been recognized as a common structural motif in intrinsically disordered peptides, such as the Aβ peptide.^208^ In this context, Dodero’s group conducted a comprehensive study of the 33-mer gliadin peptide at various concentrations and temperatures in water and citrate-phosphate saline buffer. Circular dichroism spectroscopy revealed that the peptide adopts a predominantly disordered structure with a high random coil component at low concentrations in the nanomolar range (Figure 8A). At concentrations above 197 μM, a transition toward a PPII-helix structure was observed, characterized by a subtle positive signal at 228 nm (Figure 8B). The β-structure became predominant at higher concentrations (Figure 8C). This structural shift was further confirmed by the addition of urea, which stabilized the PPII-helix conformation. Temperature-dependence experiments confirmed the equilibrium between the PPII-helix, which was predominant at low temperatures such as 5 °C (black line), and the β-structure, which became the major component at 37 °C (red line) (Figure 8A–C). The concomitant hypochromic displacement of the negative signal with increasing peptide concentration, accompanied by a minimum displacement from 203 to 215 nm, indicates a structural transformation due to a self-assembly process (Figure 8A–C). The formation of nanostructures was confirmed by EM, showing that the peptide forms oligomers and large fibrillar structures.^36^ These structural findings were further validated by ATR-IR and AFM, which demonstrated that the increase in peptide concentration led to a rise in β-parallel structure.^50^ However, the β-parallel sheets detected did not bind to ThT. A concentration-dependent analysis of the peptide by DLS indicated that the peptide forms a polydisperse system in solution. Interestingly, when the peptide solutions were filtered (0.22 μm Teflon filter), rapid self-association occurred at low concentrations, but this did not happen at 610 μM. This demonstrated that once larger structures, such as protofilaments, are formed, filtration can remove them. The results obtained in solution were confirmed by AFM analysis on mica. At low concentrations (6 μM), discrete spherical structures and clusters were detected (Figure 8D), but, while increasing the peptide concentration to 60 μM (Figure 8E), revealed oligomers and protofilaments. At 250 μM, the large structures evolved (Figure 8F), and at the higher concentration of 600 μM, interconnected filaments and plaques surrounded by smaller nanostructures were observed (Figure 8G).

**Figure 8:**
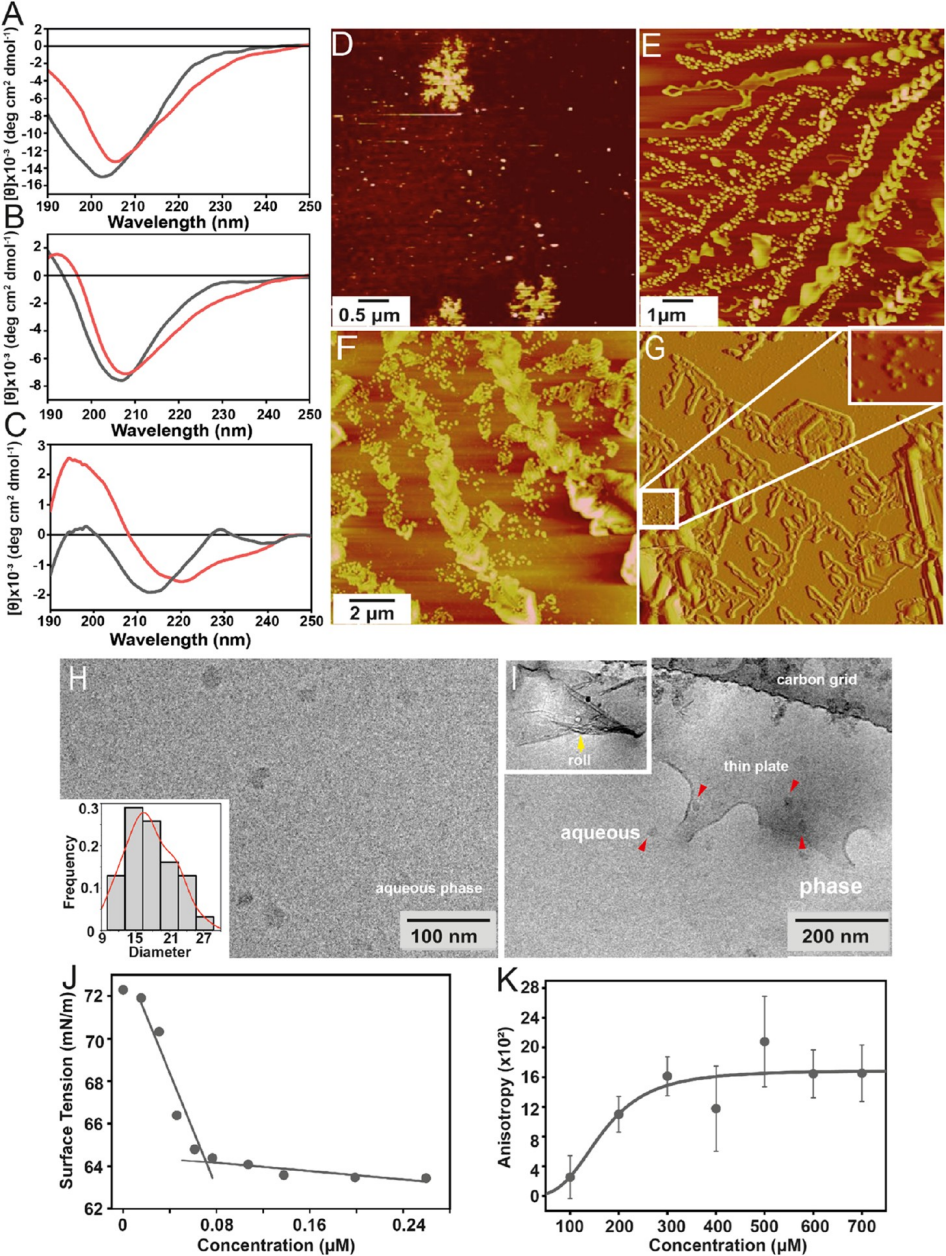
33-mer gliadin peptide self-assembles into different nanostructures depending on the peptide concentration. Circular dichroism analysis of secondary structure at (A) 6 μM, (B) 197 μM, and (C) 600 μM in Milli-Q water at −5 °C (black) and 37 °C (red).^36^ Atomic force microscopy (AFM) images at (D) 6 μM, (E) 60 μM, (F) 250 μM, and (G) 600 μM.^50^ Cryo-EM of the 33-mer peptide at 600 μM shows (H) oligomers^209^ and (I) thin-plate structures. (J) Surface tension (Plate method according to Wilhelmy) and (K) tyrosine steady-state anisotropy of the 33-mer gliadin peptide self-assembly.^209^ Reprinted with permission under Creative Commons CC BY 3.0 license from ref 209. Copyright 2022 The Authors. Published by Wiley-VCH GMbH.

To gain deeper insights into the early stages of oligomerization of the 33-mer peptide and the interactions between monomers, subsequent studies integrated imaging and biophysical techniques with molecular dynamics (MD) simulations. Herrera et al. demonstrated that, using helium ion microscopy (HIM) and scanning transmission electron microscopy (STEM), the 33-mer gliadin peptide forms spherical-like oligomers that reorganize into rod-like oligomers. These rod-like structures self-associate into protofibril-like structures by lateral association.^37^ Cryo-EM images revealed that the large structures previously detected by other microscopic techniques are large thin-plate structures that coexist with spherical-like oligomers at the higher peptide concentration of 600 μM (Figure 8H,I).^209^ To better understand the nature of this association, a surface tension analysis of the peptides at the water–air interface was conducted, showing that the 33-mer behaves as a nonionic surfactant that self-assembles above 75 nM (Figure 8J).

The characterization of thin-plate structures is particularly significant, as these larger assemblies of the 33-mer peptide appear to activate inflammatory pathways, such as the NFκB pathway, in a concentration-dependent manner in macrophage cell lines while preserving cellular viability.^37^ This report marked the first time that 33-mer nanostructures were shown to activate the innate immune response by 33-mer nanostructures for the first time. The key experiment involved a comparative analysis between unfiltered and filtered samples. As mentioned, the larger 33-mer structures can be filtered, yielding a solution mainly composed of 33-mer monomers and smaller oligomers. Stimulation with the nonfiltered sample resulted in increased secretion of IP-10 and TNF-α, cytokines that are typically upregulated in CeD patients. In contrast, the filtered samples exhibited minimal to no effect on cytokine secretion. Furthermore, it was observed that the induction of NFκB depends on the interaction of the 33-mer large structures with Toll-like receptors 2 and 4. Similar experiments in the Caco-2 cell line, a model of gut epithelial cells, showed that the 33-mer peptide can accumulate without promoting increased permeability of the cellular monolayer.^197^ In this cell line, noninflammatory markers were detected, likely because epithelial cells are less specialized immune cells than macrophages and therefore less responsive to TLR4/2 agonists.^196^

Next, a multitechnique approach was employed to comprehensively understand the initial stages of oligomerization. This study used the tyrosine (Tyr) residues in the 33-mer for intrinsic fluorescence anisotropy spectroscopy, time-resolved fluorescence, and molecular dynamics (MD) simulations. Intrinsic Tyr fluorescence analysis, including steady-state and lifetime measurements, showed that larger structures were detected above 100 μM, with no changes in the lifetimes observed above 300 μM, suggesting the stabilization of the large thin-plate structures (Figure 8K). Computational approaches, in which a dimer and decamer were analyzed, showed that Tyr residues in the 33-mer gliadin peptide slowed their rotation due to self-assembly, which aligned with the experimental results.

Considering the absence of atomic structural information, Amundarain et al. investigated the early oligomerization of the 33-mer peptide, examining dimers, trimers, tetramers, and decamers through molecular dynamics simulations. Their findings demonstrated the peptide’s propensity to self-associate into higher-order structures. Analysis of the β-sheet content in these oligomers revealed an increase in this secondary structure with larger oligomer sizes, consistent with experimental observations. Tyr-Tyr cross-linking assays, followed by SDS-PAGE, further confirmed the presence of monomers and low-molecular-weight oligomers, ranging from dimers to nonamers. Secondary structure analysis of the covalently stabilized oligomers revealed a predominant PPII-helix content. Temperature-dependent studies of these oligomers indicated a deviation from the two-state behavior observed in the non-cross-linked peptide, as evidenced by more than two isodichroic points. Nonetheless, the spectral analysis showed an increase in β-sheet content at 37 °C, which aligns with observations for the non-cross-linked peptide.^210^

The physiological relevance of these Tyr-Tyr cross-links remains unclear. Nevertheless, it has been reported that such bonds form during bread dough preparation. To the best of our knowledge, no further have investigated the role of Tyr-Tyr cross-links in gluten-related disorders. In other food products, Tyr-Tyr has been detected in milk powders and myofibrillar proteins, as well as in caseins and lactalbumin subjected to enzymatic or UV-induced oxidation. Concerns regarding dietary intake of Tyr-Tyr have prompted studies on its potential toxicity. Ingestion of pure Tyr-Tyr has been linked with metabolic disturbances, impaired glucose metabolism, and kidney alterations. Moreover, processed milk products containing Tyr-Tyr and other oxidized tyrosine derivatives have been shown to cause oxidative damage in organs such as the liver and brain, potentially impairing cognitive functions like memory and learning.^211–213^ In the case of gluten, the formation of Tyr-Tyr cross-links may add another layer of complexity to PRGPs, warranting further investigation.

A particular aspect of the 33-mer peptide is its deamidation of three glutamines into glutamic acid residues by tTG2, generating the 33-mer DGP (deaminated gliadin peptide) in active CeD. The introduction of three negative charges in 33-mer DGP affects the properties of the peptide, not only biologically, as previously described, but also structurally. Both peptides have been characterized as intrinsically disordered proteins (IDPs) in MD simulations.^214^ Herrera et al. analyzed 33-mer DGP solutions prepared as described in Table 3. Some samples were treated with HFIP, evaporated, and resuspended in water, but this treatment did not impact the obtained results. Anisotropy and surface tension measurements showed that the 33-mer DGP peptide has an amphiphilic nature and can self-associate in solution (Figure 9A,B). Detailed secondary structure analysis by CD at different peptide concentrations and pH levels showed the self-association trend observed by microscopy. The PPII-helix is the principal secondary structure adopted by the 33-mer DGP peptide, which is in equilibrium with β-sheets, as confirmed by analyzing the peptide at different temperatures and adding cosolvents (Figure 9C). AFM and TEM revealed the presence of spherical-like oligomers at different peptide concentrations, in contrast to the 33-mer peptide, which forms larger supramolecular structures as the peptide concentration increases (Figure 9D,E). MD simulations confirmed the 33-mer DGP oligomerization into small oligomers without a defined tertiary structure. Similar to what was observed experimentally, it was shown that the PPII helix is the main structural motif, with a minor proportion of β-sheet content. In a cellular context, it was observed that the 33-mer DGP accumulates inside Caco-2 cell cultures as a monolayer. However, in contrast to the 33-mer peptide, the 33-mer DGP promotes increased permeability of the monolayer, altering the localization of the tight-junction protein zonula occludens from the cell–cell contact sites to its release into the cytoplasm.^197^ In the *in vivo* context, administration of the 33-mer DGP showed rapid blood circulation and accumulation in the pancreas after 1 h of peptide administration in both healthy and diabetic mouse models.^162^ The analysis of blood samples from mice revealed the presence of large structures, which could indicate the presence of 33-mer DGP oligomers; potential modifications of the peptide in the bloodstream cannot be excluded. These are the first results on the multimerization of 33-mer DGP in an *in vivo* model and suggest that these oligomers may play a role beyond the induction of an immune response (Figure 9F).

**Figure 9:**
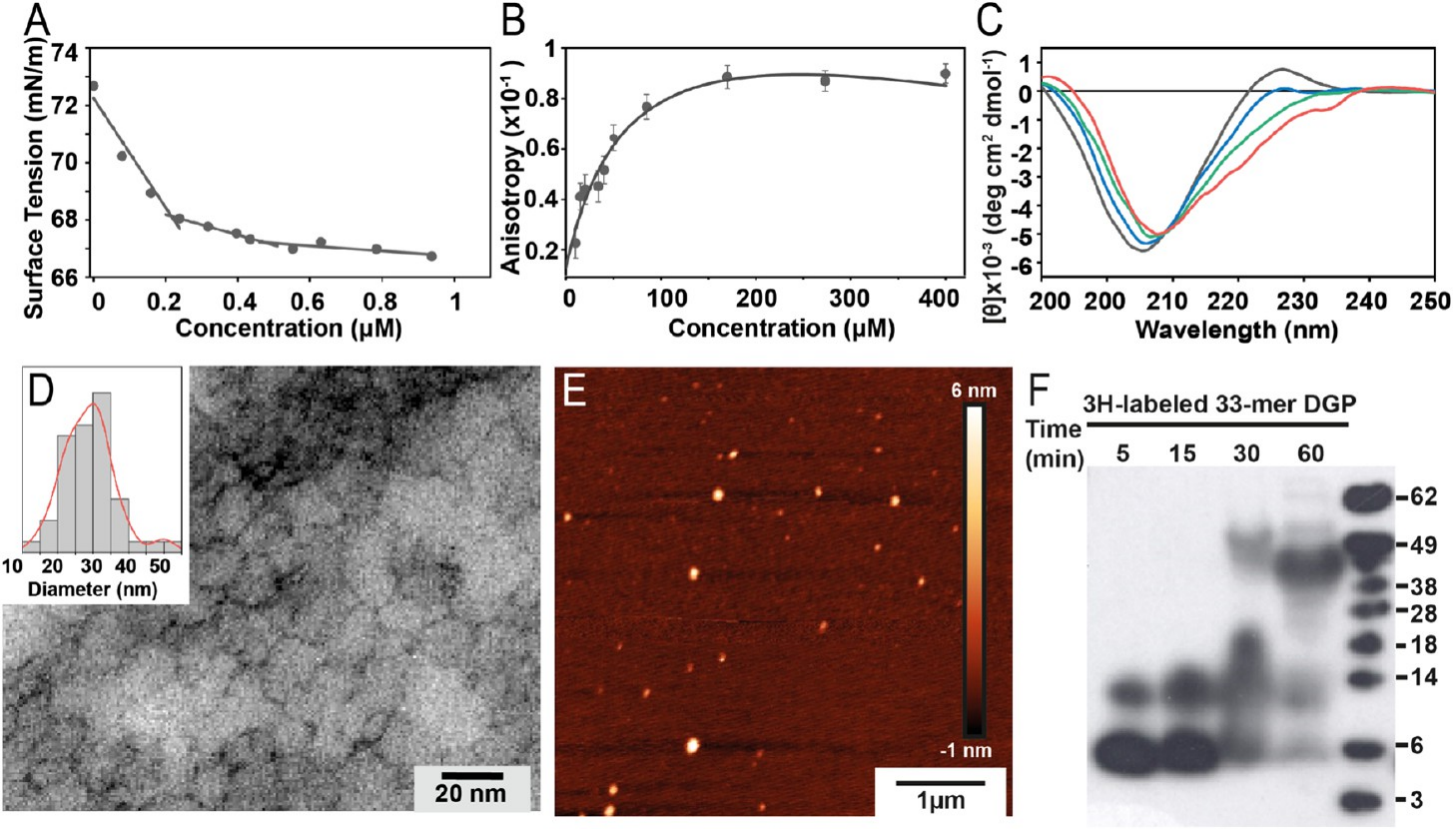
33-mer DGP self-assembles in oligomers and nanostructures. Evaluation of the self-association of the 33-mer DGP at different peptide concentrations by (A) surface tension (Plate method according to Wilhelmy) and (B) steady-state tyrosine anisotropy. (C) Circular dichroism of 33-mer DGP at 150 μM in Milli-Q water at −5 °C (black), 10 °C (blue), 25 °C (green), and 37 °C (red). Morphological analysis of the 33-mer DGP by (D) TEM and (E) AFM.^197^ (F) Kinetics of ^3^H-33-mer in mouse blood in NOD mice (6–8 weeks). Tail blood was analyzed by SDS-PAGE at the indicated time points (min).^162^ Adapted with the permission under Creative Commons CC BY 4.0 License from ref 162. Copyright 2016 The Authors. Published by Hindawi Publishing Corporation.

##### 2.2.2.2 Nanostructures of p31–43 Gliadin Peptide

The first analysis of the structure of p31–43 and its cellular effects was conducted by Vilasi et al., who used both Tyr fluorescence and the emission of a lissamine-labeled peptide in the presence of SDS-micelles to analyze the peptide. The group showed that p31–43 can interact with micelles, acting as membrane mimetics.^215^ In contrast, the short version of the 33-mer peptide (residues 56–68) cannot. Barone’s group analyzed the structure of the p31–43 peptide and two mutants (P36A and F37A) by NMR in the absence and presence of SDS micelles at pH 4.6. The p31–43 peptide adopts a PPII-helix with multiple conformational equilibria, with interconversion kinetics ranging from slow to fast on the chemical shift time scale. The NMR spectra of the peptides in water revealed more spin systems than the number of residues in their sequences. These findings suggest that two possible phenomena could be occurring: proline isomerization and a self-association process. Due to methodological restrictions, the technique could not disclose the main mechanism producing this effect. In the case of the P31A and F37A mutants, a disruption of the PPII-helix, with a higher proportion of disorder, was observed. When p31–43 is in a lipid-mimetic environment, such as in the presence of SDS micelles, it can adopt several additional structures not observed in water.^198^ However, a more detailed analysis is needed to determine the role of lipids in the structure and physicochemical properties of p31–43.

The oligomerization of p31–43 was analyzed similarly to the 33-mer peptide using various physicochemical methods. Chirdo’s group prepared the peptide in different buffers (with distinct salts and pH levels) and in water. CD spectroscopy showed that p31–43 predominantly adopts a PPII-helix structure across various pH levels and in the concentration range from 10 to 100 μM at 4 °C. At concentrations of 50 μM and 100 μM, the peptide forms spherical oligomers, which were detected by NS-EM (Figure 10A).^51^ To confirm these findings, the group performed a more detailed analysis by combining different biophysical techniques to understand the role of concentration and the temporal evolution of p31–43 self-association. Using DLS and binding assays with the oligomer-sensitive BODIPY probe, they confirmed the presence of oligomers in a concentration-dependent manner. A detailed secondary structure evaluation by CD at different peptide concentrations (10, 50, 100, 200, and 500 μM) at 4 °C revealed a hypochromic displacement of the negative band, predominantly at 200 and 500 μM, suggesting a self-assembly process. Temperature-dependent experiments, difference spectra analysis (Figure 10B), urea treatment, and TFE dilution evaluation confirmed the conformational equilibrium from PPII-helix to β-sheet structures. A detailed assessment of the morphology at different peptide concentrations and time-lapse studies was performed by AFM. At 5 μM, a polydisperse population of small spherical structures was detected; at 50 μM, larger spherical structures were observed, accompanied by some clusters. At 100 μM, mostly small-height clusters and amorphous aggregates with approximate lengths of 250 nm were observed (Figure 10C). The height of these aggregates was comparable to those observed at 50 μM, suggesting that lateral association of these nanospherical structures might occur. Time evolution analysis of p31–43 over 7 days at 10 and 50 μM showed the evolution of the system from small to larger spherical and high amorphous structures toward chain-like fractal networks and small oligomers, respectively.^199^

**Figure 10:**
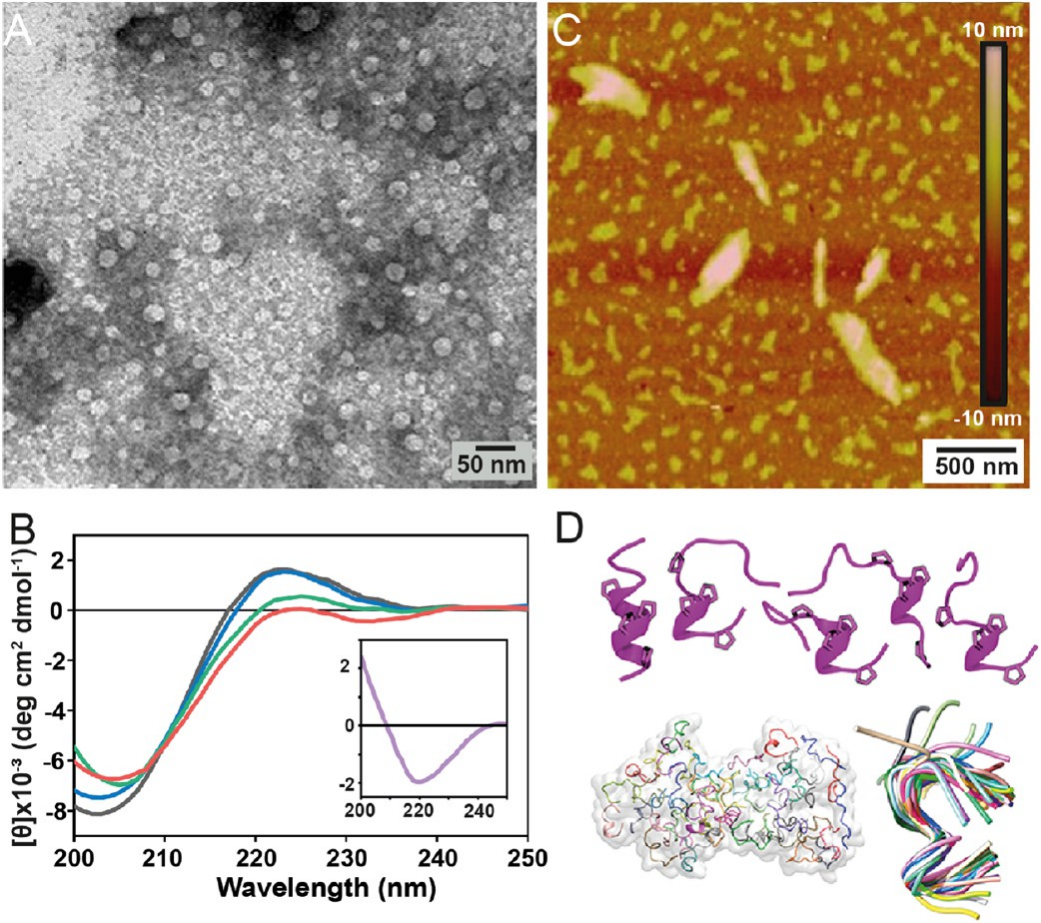
P31–43 gliadin peptide forms PPII-helix-rich spherical nanostructures and clusters. Morphological analysis of p31–43 nanostructures at 100 μM in Milli-Q water by (A) TEM^51^ and (B) Circular dichroism at 100 μM at 4 °C (black), 10 °C (blue), 25 °C (green), and 37 °C (red) in Milli-Q water. The inset presents a difference spectrum between 37 and 4 °C^199^ (C) AFM.^199^ (D) Molecular dynamics simulation of p31–43. The upper panel shows conformers arbitrarily chosen along the simulation of the isolated peptide, which was used to generate the system’s initial configuration containing 50 copies of p31–43. The lower panel shows the final state (left), with structural convergence within the aggregate highlighted by the superposition of individual copies (right).^51^ Reprinted in part with permission under Creative Commons CC BY 4.0. License from ref 51. Copyright 2019 The Authors. Published by Frontiers Media S.A.

To understand the molecular factors inducing p31–43 oligomerization, *in silico* modeling of the peptide’s structure was performed. Initially, a monomer was simulated using a PPII-helix structure as the starting point. This simulation showed that the peptide is highly flexible, except for the central proline residues P36, F37, and P38, which retained a nearly helical conformation (Figure 10D, upper panel). However, when simulating 50 copies of p31–43, spontaneous aggregation occurred through the formation of small clusters that eventually fused into a single large aggregate (Figure 10D, bottom left panel). Spontaneous dissociation events also occurred in the solution. The final MD simulation of the multimer shows that the p31–43 monomer reached structural convergence, as shown by the superposition of each monomer recovered from the final cluster in the final conformation (Figure 10D, bottom right panel). These aggregates exhibited an increase in the amount of PPII-helix compared to an isolated monomer.^51,199^

Regarding the toxicity of p31–43, several groups have investigated its impact in both cellular and *in vivo* environments, showing its ability to induce enteropathy and alter key signaling pathways of innate immunity, cell proliferation, and vesicle trafficking.^183^ Gomez-Castro et al. were the first to assess the formation of oligomers and their possible role in CeD. When p31–43 was orally administered to healthy mice, it induced intestinal damage in the proximal small intestine. This effect was associated with increased production of type I interferon and IL-1β, as well as activation of the NLRP3 inflammasome pathway, contributing to gut inflammation. These findings were an important first step toward understanding the relationship between p31–43 oligomerization and CeD-related effects.

##### 2.2.2.3 Nanostructures of Gliadin Peptides Obtained by Pepsin or Trypsin, and Pepsin–Trypsin Degradation

Considering that a complex mixture of peptides is produced during gluten digestion, the structural evaluation of gliadin digests has become an area of increasing interest in recent years. It is important to note that the preparation of gliadin digests requires the establishment of standardized methods. Recently, Xhaferaj et al. made a significant contribution to characterizing native gliadin proteins. The study revealed notable batch-to-batch differences in protein content, emphasizing the need for careful analysis to ensure consistent results in biochemical, immunological, and functional experiments. In particular, they found that gliadin content is low in some batches, while glutenins are also abundant.^216^ A widely used standard gliadin, comprising a mixture derived from 28 European cultivars, is typically referred to as gliadin PWG (prolamin working group). However, this reference is not usually used in digestion experiments.^217^

Gliadins undergo enzymatic processing, starting with pepsin digestion at low pH and 37 °C, followed by digestion at nearly neutral pH (approximately pH 7.0) or slightly basic conditions (approximately pH 8.0) for P-gliadin. Trypsin and chymotrypsin are then added, and the reaction takes at least 2–3 h to simulate the final gut digestion. Alternative protocols involve treatments with elastase and carboxypeptidase for 1 h at 37 °C, followed by incubation with brush border membrane (BBM) extracts from porcine sources for 2 h were reported.^218^ The resultant PRGP mixtures, commonly referred to PT-gliadin, can be employed or fractionated using various techniques, such as centrifugation, filtration, or even reverse-phase chromatography.^219^ For *in vivo* approaches, a further step of quantifying LPS (lipopolysaccharides) as a principal bacterial-metabolite contaminant is required. If LPS is observed, its elimination is necessary. Notably, during baking, wheat components such as gliadin undergo oxidative reactions, leading to the formation of disulfide and Tyr-Tyr bonds. These structural modifications may contribute to the diverse mix of peptides generated during digestion, potentially adding another layer of complexity to their study.^211,212,220,221^

PT-gliadin produced *in vitro* was initially studied to evaluate its cellular effects. Early studies demonstrated that PT-gliadin peptides could induce agglutination of undifferentiated cells, an effect that could be inhibited by the addition of saccharides, suggesting the involvement of polyvalent interactions.^222^ Further studies on PT-gliadin indicated that gliadin could interact with other proteins, such as bovine serum albumin, through hydrophobic interactions. These studies also suggested the self-aggregation trend of these peptides.^223^ Dias et al. prepared a PT-gliadin and separated the peptides into seven fractions using semipreparative HPLC. Upon rehydration, the freeze-dried samples formed aggregates up to 2 μm in size, as confirmed by DLS. The study also demonstrated that small molecules such as tannins can modulate the aggregation process.^203^ Similarly, Manai et al. showed that the PT-gliadin forms large aggregates detectable by fluorescent microscopy (after labeling with a fluorescent probe) in cellular media (Figure 11A).^202^ The Caco-2 cell model was able to uptake these aggregates and incorporate them into vesicles. TEM imaging further confirmed the presence of amorphous aggregates (Figure 11B).

**Figure 11:**
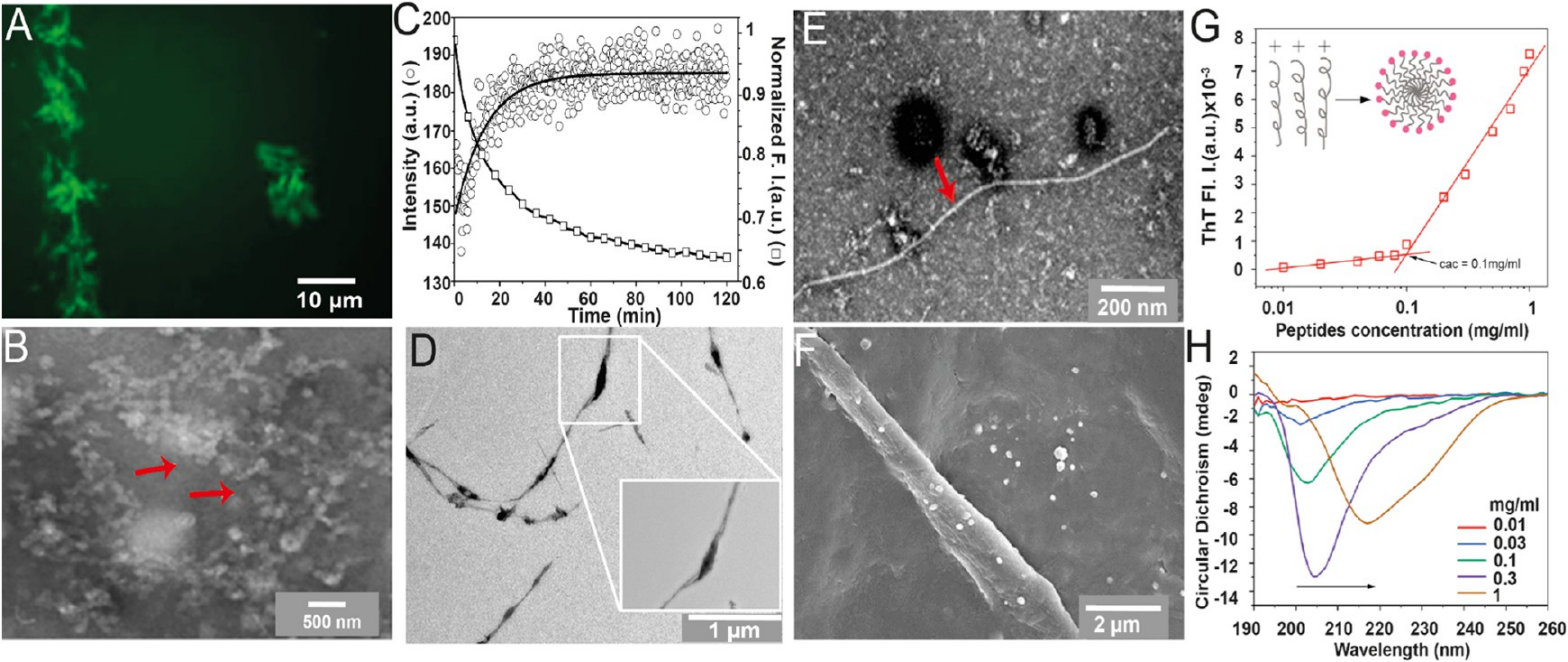
Amyloid-like fibrils formed after gliadin digestion. The PT-gliadin forms aggregates that can be observed by (A) fluorescence microscopy and (B) TEM.^202^ Reprinted in part with permission under Creative Commons CC BY 4.0. License from ref 202. Copyright 2018 The Authors. Published by MDPI. Kinetic analysis of gliadin digestion by pepsin showed the formation of aggregates and fibrils detected by (C) fluorescence and static light scattering and (D) TEM.^196^ Reprinted in part with permission under Creative Commons CC BY 4.0. License from ref 196. Copyright The Authors 2021. Published by Wiley-VCH GmbH. T-gliadin can form amyloid fibrils that are detectable by (E) TEM and (F) SEM.^200^ Reprinted in part with permission from Elsevier Science & Technology Journals ref 200. Copyright 2024 Elsevier Science. PT-gliadin cationic peptides obtained through digestion and centrifugation form structures that (G) bind to ThT in a concentration-dependent manner, with a critical aggregation concentration (CAC) around 0.1 mg/mL. (H) Concentration-dependence circular dichroism. At higher concentrations (1 mg/mL), the β-structure becomes predominant.^204^ Reprinted in part with permission from ref 204. Copyright 2021 American Chemical Society.

Previous reports showed that only the pepsin digestion of gliadin (P-gliadin) is sufficient to produce an innate immune response by activating various NFκβ signaling pathways. Herrera et al. produced proteolysis-resistant P-gliadin peptides, showing that the nanostructures spontaneously formed during proteolysis.^196^ The key experiment was performed by *in situ* static light scattering (SLS) and intrinsic fluorescence spectroscopy during pepsin proteolysis, demonstrating that P-gliadin formed structures larger than those of the initial gliadins^196^ (Figure 11C). This result was supported by calculating the aggregation index from UV–vis experiments and DLS measurements before and after 2 h of adding the digestive enzyme. The amphiphilic nature of P-gliadin was assessed through surface tension measurements, yielding a critical aggregation concentration (CAC) of 0.4 μg/mL. These findings indicated that P-gliadin aggregates above this concentration, which is much lower than the standard concentration used in *in vivo* experiments (0.5 or 1 mg/mL). The morphology of the aggregates was analyzed by TEM, revealing that oligomers and fibrils formed from P-gliadin (Figure 11D). These fibrils exhibited amyloid-like characteristics and could bind probes such as Nile red, taBODIPY, and ThT. Temperature-dependent CD experiments showed the presence of more than one isodichroic point, indicating the presence of multiple conformations.^196^

Other groups have also studied the effect of trypsin digestion on gliadin structure. Feng et al. prepared a tryptic digest of gliadin (T-gliadin) and analyzed the evolution of the peptides over time after incubation at 37 °C. This group demonstrated the formation of ThT-positive amyloid fibrils by TEM, SEM (Figure 11E,F) and DLS.^200^ The secondary structure of T-gliadin was analyzed using FT-IR, revealing the presence of stacked β-sheet structures characteristic of fibril aggregates. These structures exhibited increased hydrogen bonding during the assembly process.

Additionally, intrinsic fluorescence analysis detected changes in the tertiary structure, indicating a structural rearrangement occurring during fibrillization. Zeta potential measurements further confirmed the self-assembly of the system, as an increase in zeta potential was observed during fibril formation. Consistent with these findings, Lambrecht et al. demonstrated that amyloid fibrils could also form through the tryptic digestion of gliadin.^201^ Feng et al. also analyzed glutenin and gliadin-glutenin (1:1) tryptic digests, showing that in all cases, a characteristic β-sheet structure increased upon incubation at 37 °C. The secondary structure of T-gliadin was analyzed using FT-IR, revealing the presence of stacked β-sheet structures characteristic of fibril aggregates. These structures exhibited increased hydrogen bonding during the assembly process. Moreover, intrinsic florescence analysis detected changes in the tertiary structure, indicating a structural rearrangement occurring during fibrillization. An increase in zeta potential further confirmed fibril formation. Consistent with these findings, Lambrecht et al. demonstrated that amyloid fibrils could also be formed through the tryptic digestion of gliadin.^200^

Nanoparticles of undigested PT-gliadin peptides were isolated after several ultracentrifugation steps. These nanostructures were spherical, with an average size of 70 nm, as confirmed by DLS and TEM. The peptides obtained exhibited highly positively charged sequences that could bind to ThT. The group demonstrated a β-sheet conformational transition above the critical aggregation concentration (CAC, ∼0.1 mg/mL), as observed through ThT fluorescence and CD analyses (Figure 11G,H). Additionally, isothermal titration calorimetry and Langmuir monolayer experiments revealed that these peptides interact with DPPC/DOPE lipid membrane models in a concentration-dependent manner. At concentrations exceeding the CAC, the peptides appeared to self-assemble on the lipid membranes, forming hydrogen bonds that drive this interaction.^204^

#### 2.2.3 Key Takeaways on the Aggregation of PRGPs

The common molecular characteristic of PRGPs is their high content of proline and glutamine residues, which makes them poorly digestible by human digestive enzymes such as pepsin, trypsin, and chymotrypsin. These PRGPs can self-assemble into multimers, oligomers, and large structures: thin plates (protofibrils, ThT-negative),^28,209^ amyloid-like fibrils (short fibrils, ThT-positive),^196^ and amyloid fibrils (large fibrils, ThT-positive signal similar to those observed in Aβ peptides). Multimers and oligomers predominantly adopt a PPII-helix structure, which transitions into a dominant β-structure as they aggregate into amyloid fibrils. Structural insights into these conformational changes were primarily obtained using circular dichroism (CD), which identified both PPII-helix and β-structures, supplemented by molecular dynamics (MD) simulations.^36,197,209,210^ The formation of parallel β-sheet structures was further confirmed through ATR-IR spectroscopy.^50^ As described, the secondary structure correlates with the morphology of the nanostructures formed; for example, PPII-helix is observed in oligomers, with an increase in β-sheets when protofibrils are detected. There is high reproducibility, as different structures are kinetically trapped at a particular peptide concentration, a characteristic typical of colloids.^50,197,209^

Although only a few reports have tested the effect of PRGP nanostructures in a cellular context, it has been shown that these supramolecular assemblies can elicit various pathological processes, mainly inducing cellular stress, inflammation, innate immune responses, and increased cellular permeability.^37,51,182,183,197,198^ In the literature, the cellular effects of PRGPs have generally been tested above the obtained CAC, which means the effects of the soluble PRGP nanostructures were already observed.^173,174,177,178,186^ However, this remains speculative, and one of the key objectives of this review is to emphasize the importance of thorough biophysical characterization of PRGPs before conducting cellular experiments. Notably, when PRGPs are in a mixture, they form amyloid-like or amyloid fibrils with properties comparable to those of Aβ peptides, highlighting that, in the case of PRGPs, coassembly (probably hetero-oligomerization) is essential for amyloid formation.^196,200–204^ Further research is necessary to elucidate the mechanisms underlying PRGPs fibrillation.

## 3 Aβ-Peptide and PRGP Nanostructures: Similarities, Differences, Gaps and New Research Directions

Here, we compare both peptide families—Aβ peptides and gliadin-derived peptides—to promote cross-disciplinary exchange between research areas and extend the current understanding of peptide self-assembly to other systems. By examining their structural and functional characteristics, this comparison provides a valuable framework to uncover universal principles governing peptide behavior while highlighting system-specific differences that offer insights into their structural features, biological roles, and pathological implications.

### 3.1 One Sequence, Many Faces: the Complex Behavior of Aggregating Peptides

First, let us consider their resistance to proteolysis and self-assembly capacity. A defining feature of both peptide nanostructure families is their resistance to enzymatic degradation, regardless of whether they are endogenous (Aβ) or exogenous (gliadin peptides). On the one hand, Aβ resists clearance mechanisms in the brain when it aggregates; on the other hand, gliadin peptides are not fully digested in the gastrointestinal tract, allowing them to persist, circulate, and interact with the immune system. Beyond their primary sequences, both peptides form nanostructures that progress from soluble multimers, oligomers, and protofibrils to insoluble deposits (Tables 1–3). The formation of these nanostructures represents a hallmark of their aggregation processes and acts as a potent enhancer of their proteolysis resistance. By adopting organized, compact architectures, these nanostructures reduce the accessibility of proteolytic enzymes to cleavage sites, thereby stabilizing the peptides and prolonging their biological activity and pathogenic potential.

The aggregation of amyloid plaques in the brain is a hallmark of the AD pathomechanism; Aβ soluble oligomers are considered the most toxic nanostructures, because they disrupt synaptic function, impairing memory and cognition. Interestingly, it has also been hypothesized that late-stage fibrillar aggregates, often associated with disease pathology, may serve as a protective end point by sequestering toxic intermediates.^224^ In this context, fibril formation can be seen as an unavoidable mechanism to isolate harmful species, thereby reducing their direct cellular toxicity. The hypothesis that aggregation might act as a compensatory mechanism gains further support, considering the resistance of these peptides to proteolytic degradation. When natural degradation pathways fail, aggregation may represent an alternative method of neutralizing misfolded peptides by converting them into less reactive, stable structures.

Recent studies suggest that Aβ peptides have antimicrobial properties, functioning similarly to antimicrobial peptides (AMPs) by protecting the brain against microbial infections. This protective role is linked to Aβ’s ability to aggregate into oligomers and fibrils to effectively bind to and neutralize pathogens,^225^ Aβ, thereby preventing infections. In this context, Aβ’s aggregation, traditionally associated with AD pathology, may be considered as a defense mechanism against microbial invasion in the central nervous system. The pathogen hypothesis of AD proposes that brain infections may induce Aβ production, secretion, and aggregation as part of an innate immune response. Under these circumstances, emerging evidence underscores the relevance of the gut–brain axis in Aβ aggregation, suggesting that gut microbiota and intestinal immune signaling influence neuroinflammation and amyloid pathology.^21,22^ In some individuals, excessive Aβ accumulation surpasses clearance capacity, potentially triggering AD. Hence, the onset of AD in the elderly may be linked to weakened infection resistance and increased blood-brain barrier permeability, allowing greater pathogen entry or reactivation of latent infections.^225^ This dual perspective—viewing Aβ aggregation as both a pathological and protective process—highlights the complexity of its role in AD and the relevance of the gut-brain axis as a potential target for preventive strategies. Therapeutically, this insight suggests a focus on inhibiting early stage oligomers while potentially leveraging the sequestration role of fibrils to minimize damage. A deeper understanding of aggregation dynamics could reveal novel strategies to modulate these processes, providing new pathways to manage peptide-related diseases more effectively.

On the contrary, the knowledge of the capability of PRGP to form nanostructures is relatively new;^36,199^ therefore, their role on CeD or other GRDs is an emerging area of research, with potential implications for human nutrition that require further investigation. The amphiphilic nature of gliadin peptides and their ability to be stabilized in nanostructures at low concentrations provide valuable insights into why immune cells recognize them as a pathogenic trigger.^196,209^ Thus, prompting their uptake and activation, the similarity of PRGP sequences with known pathogens can be a strong priming for immune activation. This behavior, along with the detected deposits of TG-immunocomplexes,^186–191,193^ underscores the need for further ultrastructural characterization of gliadin aggregates in CeD. Probably, the high concentration of soluble oligomers that do not evolve to insoluble fibrils has made their characterization challenging.

Given that PRGPs are exogenous, a potential but less explored alternative is to obtain a less toxic/immunogenic wheat or promoting their degradation during bread manipulation,^226–230^ at least to reduce the high concentration of GIPs, followed by the sequestration of remaining GIPs^231^ during digestion to inhibit the formation of soluble oligomers and their entrance into circulation.

Here, it is important to note that although the human body cannot degrade PRGPs, the use of bacteria that secrete endopeptidases has been explored as a nutritional strategy. However, the formation of stable nanostructures during degradation is a potential drawback that limits their successful application.^232–235^ In such cases, further investigation is needed to understand the role of microbiota in preventing or treating GRDs and lowering the inflammatory effects of PRGPs. Finally, it could be the case that the PRGPs nanostructures possess antimicrobial properties or are necessary for maintaining gut health; therefore, investigating the necessity of such peptides and their nanostructures outside pathological contexts is needed, similar to what was has been proposed for Aβ (see above). We believe that continued efforts to identify bacteria that can degrade PRGPs in gut could be relevant in the context of detoxification of peripheral Aβ nanostructures, as well as interventions targeting the gut-brain axis.^22,43^

### 3.2 Disclosing Peptide Self-Assembly Process

Second, let us compare their structural features and self-assembly mechanisms. Aβ aggregation occurs in the unique environment of the brain, influenced by factors such as the extracellular milieu, lipid membranes, and metal ion concentrations (Table 2). In contrast, gliadin aggregation occurs in the gastrointestinal tract and immune-privileged sites, where digestive enzymes and immune surveillance play critical roles. The self-assembly of PRGPs *in vitro* is well-known, and the formation of amyloid fibrils depends on heteroassembly (Table 3). In cellular contexts, this is less studied. However, initial reports have shown that stimulation with mixtures of peptides and their supramolecular structures triggers much more severe and complex cellular responses than individual peptides, likely following synergistic pathways (Table 3). The ultrastructural characterization of Aβ peptides derived from brain fibrils has marked a significant milestone in AD research (Table 1). However, structural data obtained from patients with AD remain limited relative to the disease’s incidence, and information on the clinical history of donors is lacking. As a result, even if the correlation between a specific morphology with physiopathology has yet to be established, the structural information collected during the last years has opened new avenues for exploration. In this context, having more detailed clinical information about fibril donors would be crucial to map the physiopathology of AD. The structural analysis of end point fibrils provides more detailed insights than oligomers—due to the transient nature of the latter—focusing on the stabilization of the initial monomeric structure seems promising. A complete unfolding of the native protein precursor is required to establish irreversible amyloid fibrils with a pathologically relevant morphology. Controlling the aggregation process from the early stages preceding the misfolding step can offer a comprehensive understanding of the entire process up to the fibrils, providing crucial insights into the pathogenesis of AD.

A primary goal within the chemical community is achieving control over the monomerization of Aβ peptides, whether synthetic or recombinant. The challenge lies in the intrinsically disordered nature of Aβ peptides and the role of specific hydrophobic sequences in promoting the formation of β-sheet structures. These structures self-assemble to minimize the exposure of their hydrophobic surfaces to the solvent. However, alternative conformations, such as the transient PPII-helix and α-helices, help maintain the peptide in its monomeric state. The hypothesis that a potential transition toward a PPII-helix may participate in the transition from α helix to β-strand offers insight into the underlying mechanism of their aggregation potential.^236,237^ In this context, the self-assembly of intrinsically disordered PRGPs is different, as oligomers and protofibrils can be stabilized at a specific peptide concentration and isolated by syringe filtration for at least 2 days.^37^ At low concentrations, 33-mer and p31–43 are mainly disordered; increasing the concentration to the micromolar range favors the transition to a PPII-helix.^36,199^ Under the experimental conditions, no amyloid fibrils were detected in these two PRGPs, and the PPII-helix structure remained stable. As the peptide concentration increases, the β-content also rises, forming planar structures that represent a prefibrillar stage which activates macrophage.^37^

To obtain Aβ monomers, treatment with denaturants or solvents, such as HFIP or NaOH, helps to reduce pre-existing aggregates and initiate aggregation from a monomeric state (Section 2.1.3). In contrast, the effect of such chemicals on PRGPs, only HFIP was investigated, showing no clear changes although further investigation with other additives are required. Nevertheless, far more research is needed in both systems to standardize reconstitution protocols and achieve reproducibility across different laboratories and the summaries in Tables 2 and 3 intend to be a starting point. A novel aspect is that the posttranslational modification of 33-mer to 33-mer DGP mediated by TG2 in the gut epithelium can be a disruptor of CeD, wherein large oligomers are transformed to small oligomers with increase intestinal permeability.^197^

In both systems, it is evident that a combination of standard spectroscopy methodologies, such as CD, ATR-IR, and fluorescence, along with microscopic characterization by TEM and AFM, and, if possible, cryo-EM techniques, seems essential (Tables 2 and 3). This multimodal approach allows for a thorough analysis of the structural transformations and reaction kinetics of the self-aggregating system. By doing so, it is possible to establish clearer connections between these dynamic changes, structural polymorphs, and the various aggregation pathways they undergo than using only one technique. The β-sheet content can be monitored over time through ThT fluorescence spectroscopy, assessing the formation of Aβ amyloid aggregates in all the insoluble species (fibrils, protofibrils, and amorphous aggregates) (Tables 2 and 3). Solvatochromic dyes such as taBODIPY, however, are able to detect early and transient soluble oligomers with incipient β-sheet. In this regard, new dyes should be developed to detect nanostructures with a high content of PPII-helix. As a suitable initial protocol, we recommend in the case of the β-sheet fibrils, the ThT assay should be correlated with TEM at the final stage and check the absence of nanostructures at initial times. In the case of peptides that do not form amyloids or amyloid-like fibrils, CD is the best technique to evaluate secondary structure transitions, in time and at different environmental conditions, especially for those peptides with high PPII-secondary structure. CD is a unique technique that senses this secondary structure; the presence of nanostructures is best tested with TEM, but different staining protocols need to be checked. It is important to mention that another microscopy technique needs to validate TEM results, and here, AFM is easy. In such a case, testing different substrates is required; we recommend the use of mica as a substrate because it correlates better with TEM and cryoTEM.

## 4 Conclusions

In summary, despite their distinct origins and pathological contexts, Aβ peptides and PRGPs exhibit intriguing parallels and differences that provide compelling examples encompassing a broad spectrum of sequence variability, self-assembly mechanisms, and biological functions. At the same time, as reviewed here, both research areas highlight the significant challenges in understanding the complex relationships between structure, self-assembly, and biological/pathological activity. Thus, it requires an integrative and interdisciplinary approach, leveraging advanced biophysical, biochemical, and computational methodologies to elucidate the intricate interplay between morphology and biological function. In the case of Aβ peptides, the most studied scenario is the brain, although recent discoveries have begun to explore their role in immune responses and the brain–gut connection. For PRGPs, the primary target organ is the gut, where the adaptive immune response in CeD is well established. However, much more research is needed to understand their role in the innate immune response in the whole spectrum of GRDs. Although the gut is the main entry point for PRGPs, there is growing evidence of their biodistribution throughout the body. Nonetheless, to the best of our knowledge, any direct effect they may have on the brain remains unknown. We emphasize that understanding the structural features of the nanostructures can be the starting point to discover how to control the oligomerization in vivo in their target organ and beyond: We believe that drawing insights from both systems can aid to generate new ideas and methodologies to disclose their respective self-assembly processes and the effect of their nanostructures in the gut-brain axis. Such research efforts will advance our understanding of both pathologies and the generated knowledge may open new avenues for rationalizing therapeutic strategies targeting protein aggregation diseases.

## ○

Paderborn University, Faculty of Natural Sciences, Department of Chemistry, Warburger Str. 100, 33098 Paderborn, Germany

## Vocabulary

Peptide Oligomers: Soluble multimeric assemblies of peptides, typically smaller than 100 nm, are held together by nonspecific hydrophobic interactions and hydrogen bonds. They generally exhibit heterogeneous and transient structures, and their cytotoxicity is often linked to their specific secondary structure content.
Peptide Protofilaments: These are intermediate and partially structured aggregates formed during the transition from soluble oligomers to mature amyloid fibril deposits. Protofilaments are typically elongated; if not, they may be referred to as larger oligomers. They exhibit some β-sheet content.
Amyloid Fibrils: Highly ordered, insoluble, and filamentous aggregates formed by peptide and/or protein self-assembly. They are characterized by a cross-β-sheet structure, whose X-ray diffraction produces spacings at 4.7 and ∼10 Å. Morphologically, they are typically unbranched, with 5–15 nm in diameter, and several micrometers in length.
Seed amplification: Process mediated by a nucleation-dependent polymerization mechanism where small amounts of pre-existing aggregates serve as seeds or nuclei to accelerate the aggregation of soluble monomeric proteins or peptides into specific aggregates with a final homogeneous structure feature.
Proteinophaties: Group of diseases characterized by the abnormal accumulation, misfolding, or aggregation of specific proteins in tissues, leading to cellular dysfunction and tissue damage, also known as conformational diseases.
PPII-helix Structure: An extended left-handed helical structure that has three residues per turn with no intrachain hydrogen bonds. This secondary structure is the structural component in intrinsically disordered peptides, and its transition toward β-structures could be an early stage in amyloid formation.
Transglutaminases (TGAse): TGase-mediated cross-linking has been reported to account for forming insoluble lesions in different proteinopathies. The TGAse II is the autoantigen in celiac disease (CeD).
